# Lipidomics study of the therapeutic mechanism of Plantaginis Semen in potassium oxonate-induced hyperuricemia rat

**DOI:** 10.1186/s12906-021-03350-x

**Published:** 2021-06-25

**Authors:** Fei Yang, Wenjun Shi, Liting Wang, Nankun Qin, Chengxiang Wang, Yuying Guo, Guang Xu, Jie Fang, Xue Yu, Qun Ma

**Affiliations:** 1 School of Chinese Materia Medica, Beijing University of Chinese Medicine, Beijing, 102488 China; 2 School of Traditional Chinese Medicine, Beijing University of Chinese Medicine, Beijing, 100029 China

**Keywords:** Hyperuricemia, Lipidomics, Plantaginis Semen, Lipid metabolism disorder, Lowering uric acid

## Abstract

**Background:**

Plantaginis Semen has been widely used as folk medicine and health care food against hyperuricemia (HUA) and gout, but its pharmacological mechanism remains unclear. This study investigated the therapeutic mechanism of Plantaginis Semen extract on potassium oxonate -induced HUA rats based on a lipidomics approach.

**Methods:**

A model of HUA was established by potassium oxonate intragastric administration. 42 Sprague-Dawley (SD) male rats were randomly divided into the control group, model group, benzbromarone group (10 mg/kg) and three Plantaginis Semen groups (*n* = 7). The Plantaginis Semen groups were treated orally with Plantaginis Semen, 0.9375, 1.875  or 3.75 g/kg for 28 days. The levels of serum uric acid (UA), creatinine (Cr), triacylglycerol (TG) and tumor necrosis factor-α (TNF-α) were  measured using enzyme-linked immunosorbent assay kits. Ultra performance liquid chromatography quadrupole time of flight mass spectrometry (UPLC-Q-TOF/MS) was used for the serum lipidomics analysis, multivariate statistical analysis and independent samples t-test were carried out for the pattern recognition and characteristic metabolites identification. The relative levels of critical regulatory factors were determined by quantitative real-time polymerase chain reaction (RT-qPCR).

**Results:**

Compared with the model group, the levels of serum UA, Cr, TG and TNF-α were significantly (*p* < 0.05) decreased in benzbromarone and three Plantaginis Semen groups. With lipidomics analysis, significant lipid metabolic perturbations were observed in HUA rats, 13 metabolites were identified as potential biomarkers and glycerophospholipid metabolism pathway was  most affected. These perturbations  were partially restored via treatment of benzbromarone and Plantaginis Semen. Additionally, the mRNA expression levels of urate anion transporter 1 (URAT1) and phosphatidylinositol 3-kinase/protein kinases B (PI3K/Akt) were significantly decreased (*p* < 0.01) after treatment with benzbromarone and high dose of Plantaginis Semen.

**Conclusions:**

Plantaginis Semen had significant effects on anti-HUA, anti-inflammatory and renal protection. It attenuated potassium oxonate-induced HUA through regulation of lipid metabolism disorder.

**Supplementary Information:**

The online version contains supplementary material available at 10.1186/s12906-021-03350-x.

## Background

Hyperuricemia (HUA), the cause of gout, and is associated with cardiovascular diseases and metabolic diseases such as diabetes hypertension and dyslipidemia [1]. HUA has been observed worldwide, placing a considerable public health burden on the society [2]. There are two categories of  medications commonly used for the treatment of HUA: uricosuric agents such as probenecid and benzbromarone, and xanthine oxidase (XOD) inhibitors such as allopurinol [3]. However, allopurinol may produce severe cutaneous reactions and there are some unanswered questions about the pharmacological interactions of probenecid and the hepatotoxicity of benzbromarone [4, 5]. So alternative or complementary therapies are  are needed to reduce the risk of HUA-induced attacks of gout.

Plantaginis Semen, the dried ripe seed of *Plantago asiatica* L. or Plantago depressa Willd., is a common traditional Chinese medicine which can be used to lower serum UA [6–13]. The URAT1 and PI3K/Akt pathway are two important potential targets of Plantaginis Semen in the treatment of HUA. URAT1 plays a central role in renal urate reabsorption and Plantaginis Semen can affect the expression of URAT1 in the kidney [14–17]. The PI3K/Akt pathway is able to trigger inflammatory and kidney injuries,  impairing renal excretion of uric acid and HUA [18–24], the treatment of gouty nephropathy with Plantaginis Semen is mainly involved with the PI3K/Akt signaling pathway [25]. HUA also is related to disorders of lipid metabolism, about 60% of patients with HUA have abnormal lipid metabolism [26]. It has been proven that Plantaginis Semen could improve lipid metabolism [27–29]. Although there has been some research, it remains necessary to conduct in-depth studies on the pharmacological effects of Plantaginis Semen in treating HUA and its potential mechanism.

Lipidomics emphasizes the identification of lipid molecular composition in cells, biofluids, tissue, or whole organism and can reflect the changes of lipid metabolism in response to perturbations or stimulations [30, 31]. Lipidomics will assist us to more deeply understand the pathological process of HUA and could be a puissant tool to explore the anti-HUA mechanism of Plantaginis Semen.

In this study, we investigated pharmacological effects of Plantaginis Semen and explored molecular mechanisms of Plantaginis Semen in treating HUA. Our findings preliminarily interpreted the relationship between lipid metabolism and HUA and supplied evidences that Plantaginis Semen may be used for the treatment of HUA in the clinic.

## Methods

### Chemicals and reagents

Benzbromarone tablets (50 mg/tablet) were provided by Excella GmbH & Co.KG (Nurnberger, Germany). Potassium oxonate was acquired from Shanghai Macklin Biochemical Co., Ltd. (Shanghai, China). Pentobarbital sodium was provided by Tianjin Fuchen Chemical Technology Co., Ltd. (Tianjin, China). LC-MS grade acetonitrile, formic acid, ammonium formate, isopropyl alcohol, methanol were supplied by America Thermo Fisher Scientific Co., Ltd. (Massachusetts, America). Analytical grade ethanol was purchased from America Thermo Fisher Scientific Co., Ltd. (Massachusetts, America). Ultrapure water was made by America Millipore Co., Ltd. Milli-q ultra pure water machine (Massachusetts, America).

### Preparation of plantaginis Semen extract

Plantaginis Semen was purchased from Tongrentang Chinese Medicine Company, (Beijing, China, catalogue number:800110551). 100 g Plantaginis Semen was taken and 65% ethanol of 8 times the amount of herbs was added to heat and reflux for 3 times, 2 h each time. The filtrate obtained from three reflux times was mixed and concentrated to 100 mL, containing crude drug content of 1.0 g·mL^− 1^. The filtrate was refrigerated for later use.

### Animal care and experimental design

Specific pathogen free (SPF) grade SD 6 weeks male rats (200 ± 20 g) were bought from Vital River Laboratory Animal Technology Co., Ltd. (Beijing, China) and the certificate number is SCXK (Jing) 2016–0006. Rats were fed with a standard laboratory environment (humidity 40–70% and temperature 20–25 °C) and hold on a 12 h/12 h light/dark cycle during the whole period. All experimental protocols were approved by the ethics committee of Beijing University of Chinese Medicine (with Ethical code: BUCM-4-2,020,111,102-4061).

After the acclimation period, 42 rats were randomly separated into six group (*n* = 7), including control group (C), model group (M), benzbromarone group (Y), high dose group (CH), medium dose group (CM) and low dose group (CL) of Plantaginis Semen. Random numbers were generated using Microsoft Excel. Because of the high oral bioavailability, the rat HUA model was established by potassium oxonate intragastric administration. At 1 h before drug administration, the rats were given potassium oxonate by intragastric administration according to 1.5 g•kg^− 1^ dose, the C group was given the corresponding volume distilled water by intragastric administration. Then the CH, CM, and CL groups were orally administered three dosages of Plantaginis Semen (0.9375 g/kg, 1.875 g/kg, 3.75 g/kg). The clinical dosage of Plantaginis Semen in Chinese Pharmacopoeia (Ch P) is 9 ~ 15 g/day [32]. The dose of Plantaginis Semen in rat is calculated by multiplying the human dose (9 g/day = 0.15 g/Kg, 15 g/day = 0.25 g/Kg) by the conversion factor 6.25 [33], the result is 0.9375 ~ 1.5625 g/Kg. In pilot study, we studied the hypouricemic effect of Plantaginis Semen at various dose including 0.47, 0.9375, 1.875 and 3.75 g/Kg, and at these 4 concentrations, Plantaginis Semen all showed the effect of lowering UA. In consideration of cost, we used 0.9375, 1.875 and 3.75 g/kg in the study. The Y group was treated with benzbromarone (10 mg/kg) once a day for 28 days. Blood and kidney were collected and prepared for ELISA and RT-qPCR. Test time was between 10 am to 6 pm and testing order was randomized daily. After the experiment, the rats were sacrificed by dislocation of spine, which approved by the animal care and use committee.

### Serum biochemistry analysis

On the 28th day of modeling, rats were anesthetized with 2% pentobarbital sodium (0.3 mL/100 g, intra-peritoneally). Blood samples were taken from the abdominal aorta by a vacuum blood collection tube, and centrifuged for 10 min (3000 rpm, 10 °C). Serum samples were stored at − 80 °C for analysis. The levels of serum UA, Cr, TG and TNF-α were measured using commercially available kits (Jiancheng, Nanjing, China) according to the manufacturer’s instructions.

### Serum UPLC-Q-TOF/MS analysis

Serum samples were thawed at room temperature before data acquisition. Protein precipitation was performed by adding 320 μL solvent mixture (chloroform/methanol 3:1,V/V) to 80 μL serum in 1.5 mL eppendorf tubes. The mixture was centrifuged at 10,000 rpm for 10 min at room temperature. The supernatant was taken for UPLC-Q-TOF/MS analysis.

The UPLC column was ACQUITY CSH C18(2.1 × 100 mm,1.7 μm, Waters Corp., Milford, MA, USA) with the column temperature maintained at 40 °C and the flow rate was set at 0.3 mL•min^− 1^. The mobile phase A is 10 mM Ammonium acetate in acetonitrile/water/ formic acid (60:40:0.1, v/v/v), mobile phase B is 0.1% formic acid in isopropanol/acetonitrile (90:10, v/v). The gradient elution programme was as follows: 0.0 ~ 3.1 min 60 ~ 57% A, 3.1 ~ 4.1 min 57 ~ 30% A, 4.1 ~ 4.3 min 30 ~ 27% A, 4.3 ~ 8.0 min 27 ~ 23% A, 8.0 ~ 8.1 min 23–60% A, 8.1 ~ 10.0 min 60% A. Every 2 μl sample solution was injected for each run.

MS analysis was performed by Xevo G2-S Q/TOF MS (Waters Corp., Milford, MA, USA) system. The ionization mode is electrospray ionization and was set in positive modes. The mass conditions were as follows: the capillary and cone voltage were set at 2.5 KV and 30 V, the source temperature was 120 °C, the desolvated gas flow was 800 L/h at a temperature of 400 °C and the cone gas flow was 20 L/h. MS data were collected in the full scan mode from m/z 50–1200 amu. Both UPLC and MS conditions refer to the previous research of group [34].

### Data processing

The raw data files acquired from the UPLC-Q/TOF-MS measurements were normalized using Micromass MarkerLynx Applications Manager version 4.1 software (Waters Corporation, MA, USA). This software can automatically complete noise filter, peak identification, peak matching and normalization under optimized parameters. The parameters of process were shown as follows: mass window is 20 mDa, retention time window is 0.50 min, marker intensity threshold is 10,000 counts, peak width at 5% height is 1.00s, noise elimination level is 6.00 [34]. Subsequently, an integrated metabolite data matrix as Excel spreadsheets composed of m/z, retention time and the corresponding peak area was generated. The normalized dataset was imported into SIMCA 13.0 (Umetrics, Sweden) for multivariate statistical analysis and SPSS Statistics 21.0 for univariate data analysis. The multivariate analysis results are expressed in the form of scores plot to observe the global clustering trends of various groups and visualize their distributions. The model parameters including R^2^ (goodness of fit) and Q^2^ (goodness of prediction) calculated from the PCA and OPLS-DA models were used to evaluate the quality of models. Finally, potential biomarkers were filtered by the results of variable importance for the projection (VIP) values (VIP > 1) and t-test values (*p* < 0.5). The above-mentioned biomarkers are deteced by Mass Fragment software combined with HMDB [35] and Lipid Maps [36] database. First, input the precise molecular mass, ionization method, and adduction information of potential biomarkers into HMDB and Lipid Maps, in accordance with the rule that the deviation of the mass-to-charge ratio (m/z) value does not exceed 0.02, the search results are verified by combining the exact number of charges and the ionization method that meet the experimental conditions. Secondly, compare the primary and secondary mass spectra of the potential biomarkers with the theoretical fragments of the HMDB search results, then infer the structure of the compound and the attribution of the fragments to obtain the HUA biomarkers. Finally, the pathway analysis of potential biomarkers and establishment of correlation metabolic networks were performed with Metabo Analyst [37] and KEGG database [38].

Measurement data are expressed by mean ± standard deviation (SD). SPSS 21.0 is used for data processing and statistical analysis. The comparison of means among multiple groups is performed by one-way analysis of variance (ANOVA). When the homogeneity of variance assumptions was satisfied, pairwise comparison between groups is performed by Tukey test, otherwise, nonparametric tests was used., *P* < 0.05 were considered as statistically significant. The graphs were drawn with GraphPad Prism 8.0.1.

### RT-qPCR

RNA from kidney tissue was extracted by Hipure Total RNA Mini Kit (Magen, Guangzhou, China). RT-qPCR was carried out using TB Green Primix Ex TaqII kit (Takara, Kyoto-fu, Japan) in Bio-Rad CFX96 Real Time PCR System (BIO-RAD, Shanghai, China). The primers used in the study are: URAT1, Forward: 5′ − CTCTGCTGGTGTATGGAGTGG-3′, Reverse: 5′ − TTTCTGGATGTCTTGGATGGT-3′, PI3K, Forward:5′ − GGTTCTTGCGAAGTGAGATAGCCC-3, Reverse:5′ − ACCTGCTGCGTGAAGTCCTGTA-3′, Akt, Forward:5′ − TGTCTCGTGAGCGCGTGTTTT-3′, Reverse:5′ − CCGTTATCTTGATGTGCCCGTC-3′. Ct (cycle threshold) value was collected. Detected in triplicate and the relative expression levels of genes were determined by the 2^−△△Ct^ method.

## Results

The analyses of rat serum biochemistry were undertaken to evaluate the lowering UA, anti-inflammatory and renal protection effects of Plantaginis Semen. Lipidomics analysis was undertaken to evaluate the lipid metabolic profile of rats, and the mRNA expression of PI3K/Akt and URAT1 were determined to explore the UA lowering mechanism of Plantaginis Semen.

### Plantaginis semen decreased the level of serum UA in HUA rats

**Fig. 1:**
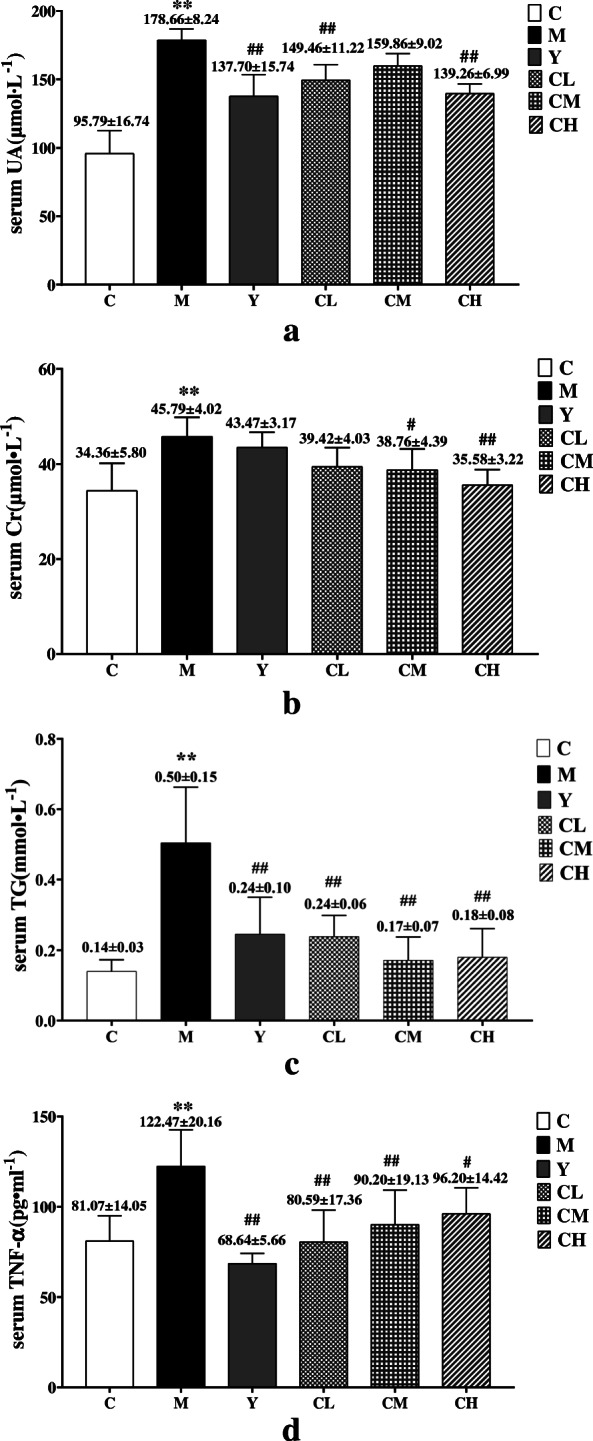
The results of serum biochemistry analysis. **a** UA level, **b** Cr level, **c** TG level, **d** TNF-α level. C, control group; M, model group; Y, benzbromarone group; CL, low dosage group; CM, medium dosage group; CH, high dosage group; values are given as the mean ± SD (*n* = 7), ANOVA, Tukey post hoc and nonparametric test were used for statistical analysis, **, *P < 0.01* vs. control group. *, P < 0.05 vs. *control group; ##, P < 0.01* vs. *model group; #, P < 0.05* vs. *model group*

After 28 days, rats fed potassium oxonate showed a significant change in the level of serum UA (Fig. 1a). As compared to the C group (95.79 ± 16.74 umol•L^− 1^), the level of serum UA in the M group (178.66 ± 8.24 umol•L^− 1^) increased significantly (*p* < 0.01), which represents the success of the model. Conversely, the level of serum UA in the Y (137.70 ± 15.74 umol•L^− 1^), CL (149.46 ± 11.23 umol•L^− 1^) and CH (139.62 ± 6.99 umol•L^− 1^) groups was significantly (p < 0.01) reduced except CM group (159.86 ± 9.02 umol•L-1, p>0.05), compared to the M group. The serum UA level of HUA rats could not be significantly (p>0.05) reduced in CM group, in addition, there were no significant differences of lowering UA effect between CL and CH group (p>0.05, supplementary material, Table 1.4). These results indicated that Plantaginis Semen could lower serum UA level.

### Plantaginis Semen exerted renal protection effect in HUA rats

Serum Cr was used to assess kidney function and reflects the extent of renal injury [39]*.* The results depicted in Fig. 1b show that the level of serum Cr in the M group (45.79 ± 4.02 μmol•L^− 1^) was significantly (*p* < 0.01) increased, as compared to that observed for the C group (34.36 ± 5.80 μmol•L^− 1^). After receiving treatment for 28 days, the serum Cr level in the CM (38.76 ± 4.39 μmol•L^− 1^) and CH (35.58 ± 3.22 μmol•L^− 1^) groups was significantly (*p* < 0.05) reduced, but the serum Cr level in the CL (39.42 ± 4.03 μmol•L^− 1^) and Y (45.79 ± 4.02 μmol•L^− 1^) groups were insignificantly (*p*>0.05) reduced compared with M group. The serum Cr level of HUA rats could not be significantly (*p*>0.05) reduced in CL group, in addition, there were no significant differences of lowering Cr effect between CM and CH group (p>0.05, supplementary material, Table 2.4). The above results indicated that the medium and high doses of Plantaginis Semen had good protective effect on renal.

### Plantaginis Semen ameliorated serum TG accumulation in HUA rats

The level of Serum TG was remarkably (*p < 0.01*) elevation in the M group (0.50 ± 0.15 mmol/L) as compared with C group (0.14 ± 0.03 mmol/L). Then, over 28 days of treatment, the TG levels in the Y (0.25 ± 0.10 mmol/L), CH (0.18 ± 0.08 mmol/L), CM (0.17 ± 0.07 mmol/L), and CL (0.24 ± 0.06 mmol/L) groups all were significantly (*p < 0.01*) reduced compared to that in the M group. The results were depicted in Fig. 1c. The results of multiple comparisons showed that there were no significant differences in the serum TG level among CL, CM and CH groups (p>0.05, supplementary materia, Table 3.4). Plantaginis Semen had good effect on lowering blood lipids.

### Plantaginis Semen reduced the level of TNF-α in HUA rats

The level of TNF-α in the serum was used to evaluate the anti- inflammatory effect of Plantaginis Semen in vivo. As shown in Fig. 1d, the level of TNF-α in the M group (122.47 ± 20.16 pg•mL^− 1^) significantly (p < 0.01) higher than that in the C group (81.07 ± 14.05 pg•mL^− 1^). The level of TNF-α in Y group (68.64 ± 5.66 pg•mL^− 1^), CL group (80.59 ± 17.63 pg•mL^− 1^), CM group (90.20 ± 19.13 pg•mL^− 1^) and CH group (96.20 ± 14.42 pg•mL^− 1^) were significantly decreased compared to M group (*p < 0.05)*, and Tukey post hoc showed that there were no significantly differences in the level of TNF-α among CL, CM and CH groups (*p*>*0.05,* supplementary material, Table 4.4). These results indicated that Plantaginis Semen had good anti-inflammatory effect.

### Metabolic perturbations and differential metabolites associated with HUA rats

**Fig. 2:**
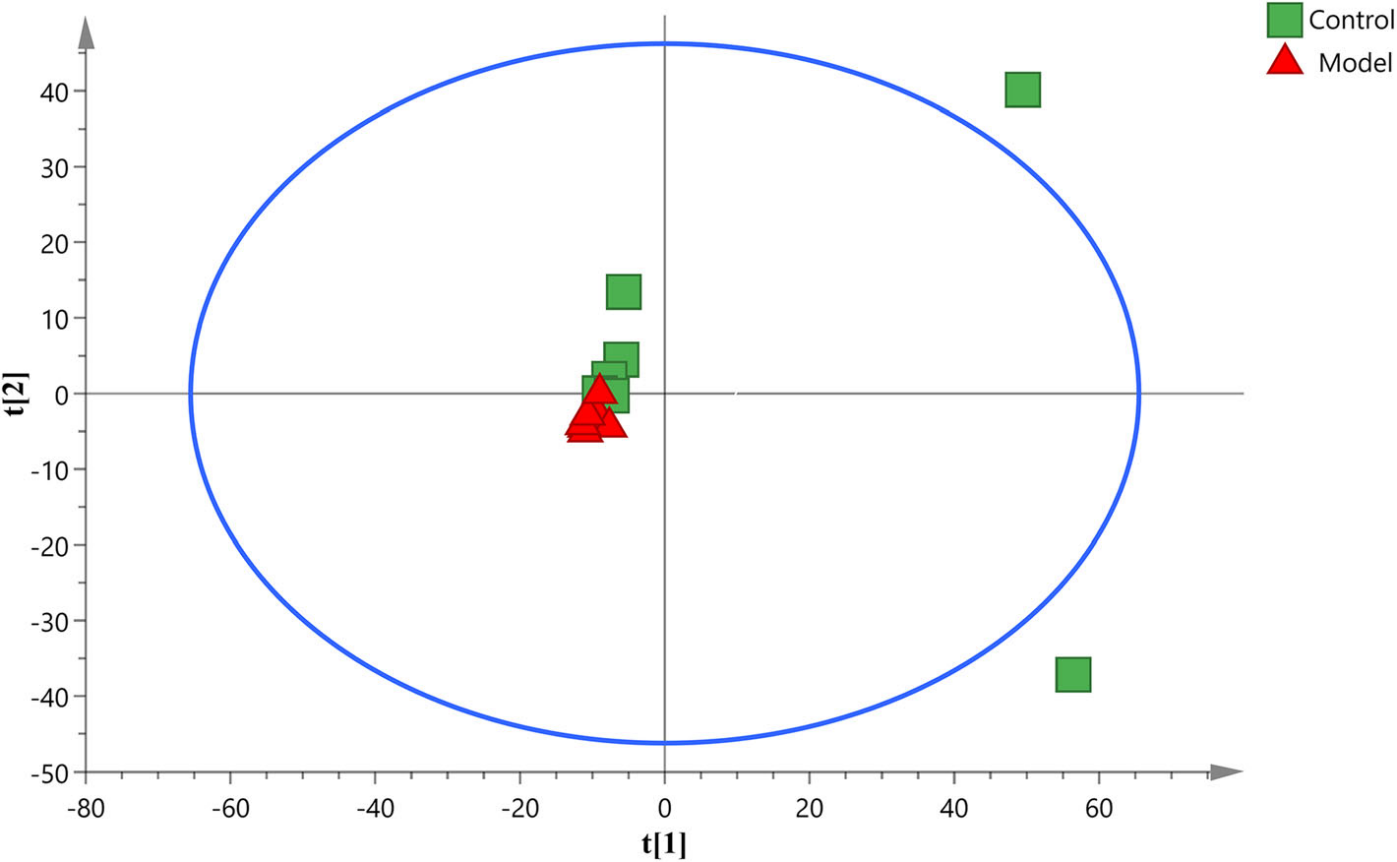
The PCA score plot derived from UPLC-Q-TOF/MS profiles of serum sample from control group and model group

**Fig. 3:**
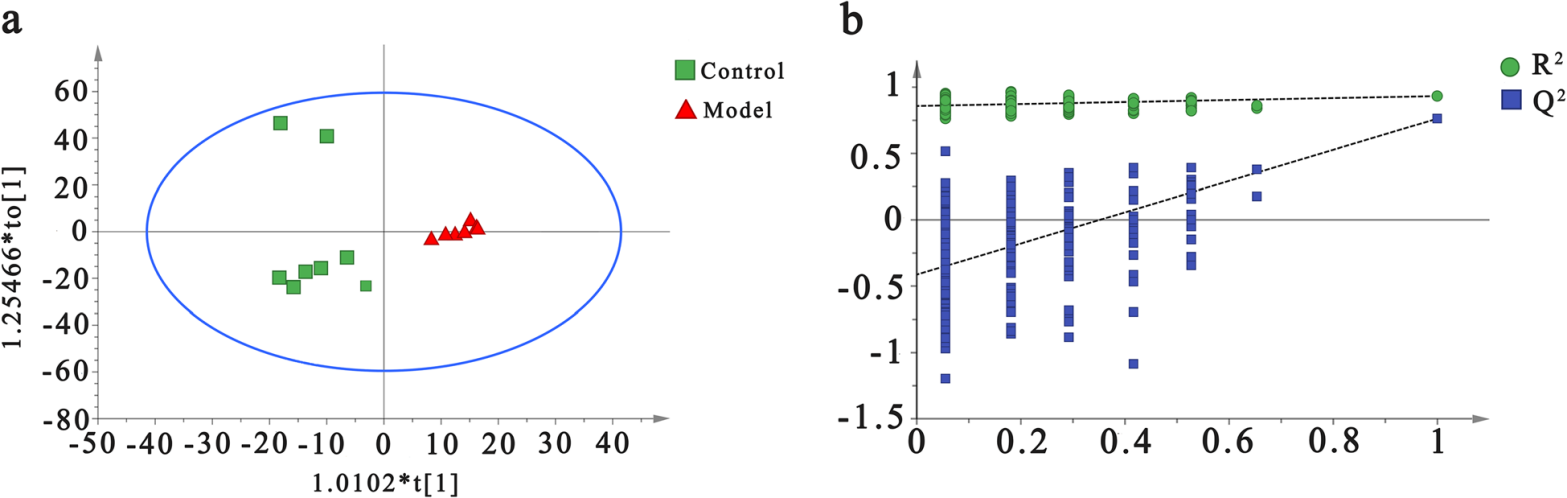
OPLS-DA score plots (**a**) and the corresponding validation plots (**b**) with 200 times permutation tests obtained

PCA is an unsupervised pattern recognition method that can be used to select different variables and find possible biomarkers, in order to investigate the effect of potassium oxonate on endogenous components changes, PCA was used to perform unsupervised data analysis on control and model groups (Fig. 2). These groups cannot be easily distinguished from each other. Therefore, we further used OPLS-DA to compare the serum samples obtained from the model and control groups. Import the normalized dataset of C and M group into the SIMCA, as shown in the OPLS-DA score plots of serum samples (Fig. 3a), a separation between the model group and the control group could be clearly seen, indicating that the HUA model was successful and had a completely different metabolic profiles compared with the healthy controls. The parameters of the OPLS-DA models were as follows: R^2^Y = 0.936 and Q^2^ = 0.737. The R^2^Y and Q^2^ values reflect excellent predictability and explain the differences between the control and model groups. In addition, 200-iteration permutation tests were also performed to assess the robustness of the OPLS-DA models (Fig. 3b). The validation plots showed that the original OPLS-DA models were not random and overfitted as both permutated Q^2^ and R^2^ values were lower than the corresponding original values along with the Y-intercepts of the regression lines of the Q^2^-points below zero.

Potential biomarkers were identified from the interactions between control and model groups using the corresponding S-plot analysis under OPLS-DA model. The metabolites whose VIP values> 1 and *p* values< 0.05 of OPLS-DA were presumed as significant differences. Databases such as KEGG and HMDB were used to identify potential bio-markers, along with UPLC-Q-TOF/MS information. The results are shown in Table 1. 13 metabolites were identified, including 4 phosphatidylcholines (PCs), 4 haemolytic phosphatidylcholines (LPCs), 2 phosphatidylethanolamines (PEs), 2 TGs, 1 cholesterol ester (CE). Compared with C group, the metabolic perturbations occurring in serum of the HUA rats were mainly characterized by increased levels of these lipids.

**Table 1:** Identification results of differential metabolites and their change trends

No.	RT(min)	*m/z*	Formula	Metabolite	VIP	M vs. C	Y vs. M	CL vs. M	CM vs. M	CH vs. M
1	5.5875	898.7862	C_57_H_100_O_6_	TG(18:2/18:1/18:2)	2.75	↑**	–	–	↓#	↓##
2	8.9231	931.7526	C_55_H_92_O_6_	TG(20:4/14:0/18:3)	2.55	↑**	–	–	–	–
3	8.4467	925.7098	C_48_H_92_NO_8_P	PC(24:1/16:1)	2.54	↑**	–	–	↓#	–
4	2.6081	496.3364	C_50_H_94_NO_8_P	PC(24:1/18:2)	2.43	↑**	–	–	↓##	↓#
5	9.0509	264.9627	C_41_H_82_NO_8_P	PC(15:0/18:0)	2.29	↑**	–	–	↓#	–
6	9.0937	326.9082	C_54_H_102_NO_8_P	PC(24:1/22:2)	2.70	↑**	↓##	–	↓##	↓##
7	1.8681	516.3129	C_24_H_48_NO_7_P	LPC(16:1/0:0)	2.35	↑**	–	–	–	–
8	2.0389	610.3188	C_30_H_54_NO_7_P	LPC(22:4/0:0)	2.56	↑**	–	↓#	–	–
9	2.0460	542.3279	C_26_H_50_NO_7_P	LPC(18:2/0:0)	2.37	↑**	–	↓##	–	–
10	4.1085	273.6606	C_26_H_54_NO_7_P	LPC(18:0/0:0)	2.09	↑*	–	–	–	–
11	9.3573	416.2983	C_45_H_76_NO_8_P	PE(18:3/22:4)	2.15	↑**	–	–	↓#	↓#
12	9.0937	326.9082	C_53_H_102_NO_8_P	PE(24:1/24:1)	2.43	↑*	–	–	↓##	↓##
13	9.0230	376.8943	C_51_H_90_O_2_	CE(24:1)	2.60	↑*	↓#	–	↓##	↓##

C, control group; M, model group; Y, benzbromarone group; CL, low dosage group; CM, medium dosage group; CH, high dosage group; **, *p <* 0.01 vs. control group. *, *p <* 0.05 vs. control group; ##, *p < 0*.01 vs. model group; #, *p < 0.*05 vs. model group. (↑): up-regulated and (↓): down-regulated. (−):no statistically significant difference

### Metabolic changes under the treatment of benzbromarone and Plantaginis Semen

**Fig. 4:**
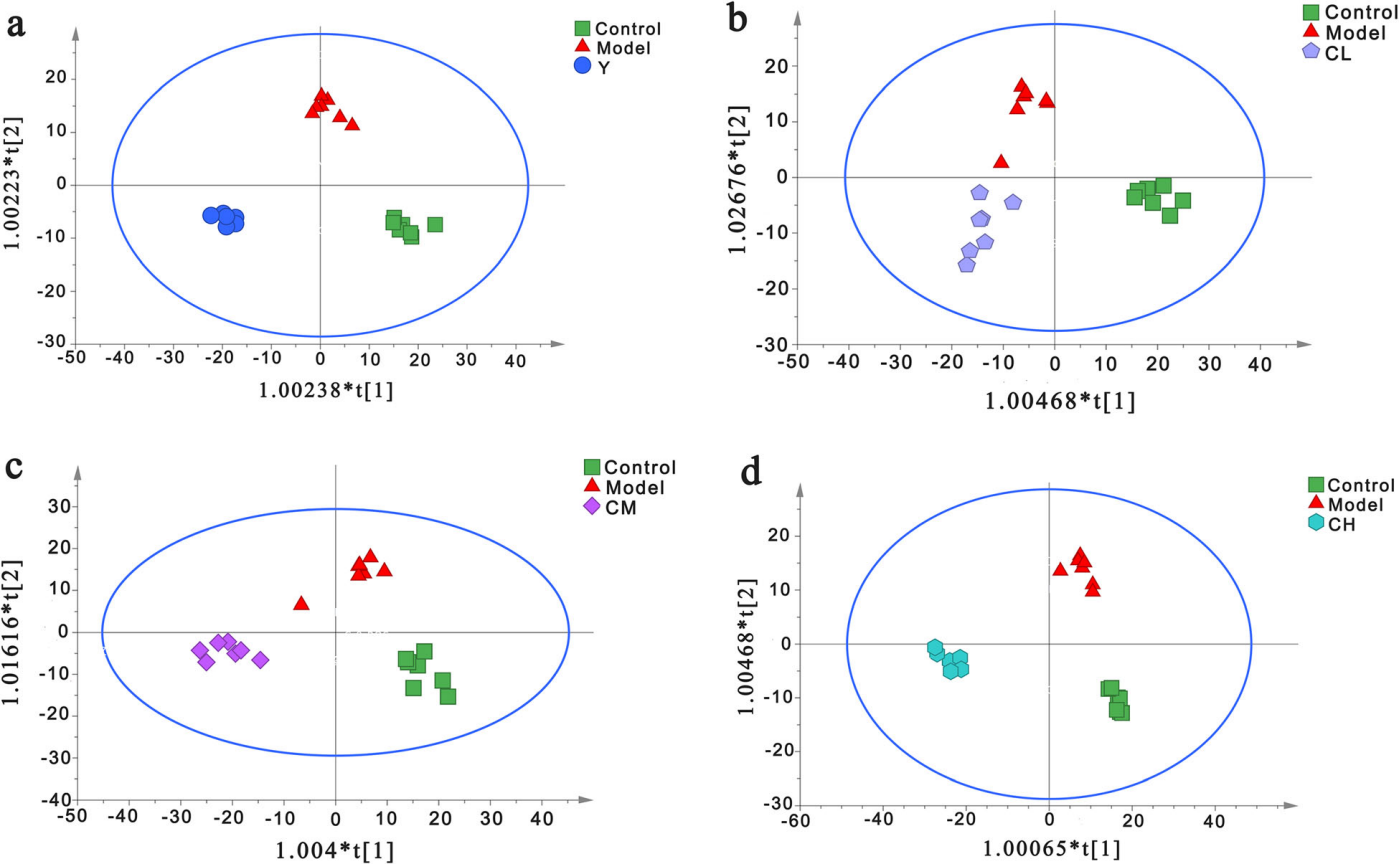
Metabolic profiles of rat serum in the control, model, benzbromarone, and different dose Plantaginis semen groups. **a** Metabolic profiles of rat serum in C, M and Y group, **b** Metabolic profiles of rat serum in C, M and CL group, **c** Metabolic profiles of rat serum in C, M and CM group, **d** Metabolic profiles of rat serum in C, M and CH group. C, control group; M, model group; Y, benzbromarone group; CL, low dosage group; CM, medium dosage group; CH, high dosage group

**Fig. 5:**
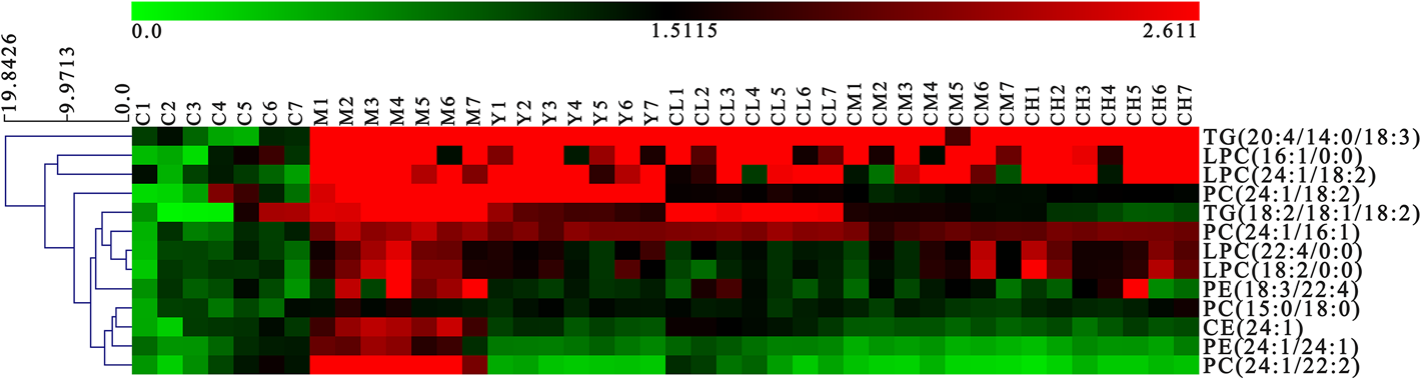
Heat map analysis of relative contents of potential metabolites. (green through dark red corresponding to a progressive increase in concentration). C, control group; M, model group; Y, benzbromarone group; CL, low dosage group; CM, medium dosage group; CH, high dosage group

**Fig. 6:**
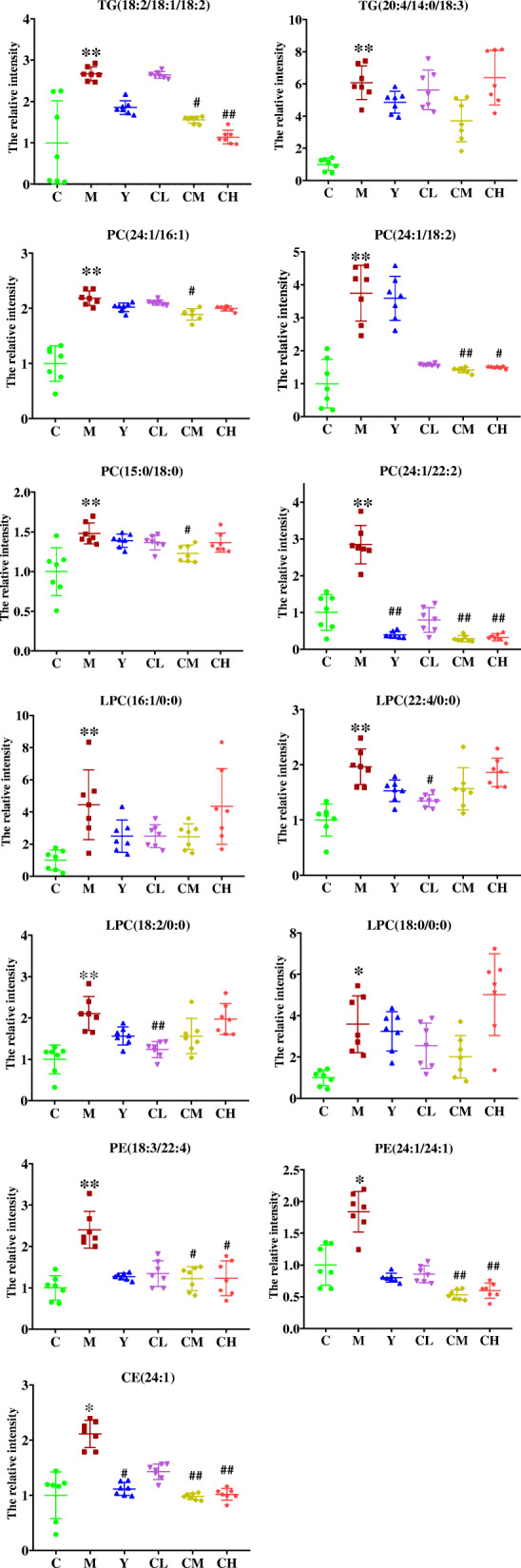
Comparison of 13 biomarkers peak relative signal intensities in 6 groups. C, control group; M, model group; Y, benzbromarone group; CL, low dosage group; CM, medium dosage group; CH, high dosage group; boxplots show the 25, 50 and 75% percentiles. ANOVA, Tukey post hoc and nonparametric test were used for statistical analysis, **, *P < 0.01* vs. control group. *, P < 0.05 vs. *control group; ##, P < 0.01* vs. *model group; #, P < 0.05* vs. *model group*

We import the normalized dataset of C group, M group and other 4 experimental groups into the SIMCA separately, Fig. 4 showed distinct metabolic profiles among different groups and there is a tendency to return to the normal group in benzbromarone-treated and different dose Plantaginis Semen-treated groups. The parameters of the OPLS-DA models were as follows: R^2^Y = 0.979 and Q^2^ = 0.830 (Fig. 4a), R^2^Y = 0.911 and Q^2^ = 0.709 (Fig. 4b), R^2^Y = 0.930 and Q^2^ = 0.781 (Fig. 4c), R^2^Y = 0.977 and Q^2^ = 0.829 (Fig. 4d). Heatmap analysis was produced to intuitively compare the relative content of 13 potential metabolites among 6 groups referring to Table 1 (Fig. 5). Control group and model group clearly distinguished, the color depth of benzbromarone group and Plantaginis Semen groups were close to control group, indicating that the performance on the callback of these metabolites is extraordinary obvious. 2, 2, 8 and 6 differential metabolites were significantly (*p < 0.*05) reversed by benzbromarone, low dose Plantaginis Semen, medium dose Plantaginis Semen, high dose Plantaginis Semen respectively (Fig. 6). Relative intensities of 13 differential metabolites in serum samples of 6 groups are listed in Table 2, the specific *p*-values are provided in the supplementary material Table 8-Table 20. These findings suggested that the metabolic perturbations induced by HUA could be normalized by benzbromarone and Plantaginis Semen treatment. Among them, the effect of normalizing differential metabolites of middle dose Plantaginis Semen group was the best.

**Table 2:** Relative intensities of 13 differential metabolites in serum samples of 6 groups

Differential metabolites	C	M	Y	CL	CM	CH
TG(18:2/18:1/18:2)	1.00 ± 1.01	2.67 ± 0.17**	1.85 ± 0.16	2.64 ± 0.08	1.56 ± 0.08**#**	1.14 ± 0.17**##**
TG(20:4/14:0/18:3)	1.00 ± 038	6.08 ± 1.05**	4.86 ± 0.68	5.63 ± 1.23	3.71 ± 1.30	6.39 ± 1.68
PC(24:1/16:1)	1.00 ± 0.32	2.18 ± 0.13**	2.02 ± 0.08	2.11 ± 0.45	1.89 ± 0.11**#**	2.00 ± 0.04
PC(24:1/18:2)	1.00 ± 0.73	3.78 ± 0.85**	3.59 ± 0.67	1.57 ± 0.34	1.41 ± 0.79**##**	1.50 ± 0.03#
PC(15:0/18:0)	1.00 ± 0.30	1.48 ± 0.13**	1.39 ± 0.08	1.36 ± 0.09	1.23 ± 0.10**#**	1.37 ± 0.12
PC(24:1/22:2)	1.00 ± 0.48	2.85 ± 0.52**	0.39 ± 0.09**##**	0.79 ± 0.33	0.29 ± 0.08**##**	0.32 ± 0.10**##**
LPC(16:1/0:0)	1.00 ± 0.62	4.45 ± 2.16**	2.49 ± 1.00	2.49 ± 0.71	2.46 ± 0.80	4.34 ± 2.35
LPC(22:4/0:0)	1.00 ± 0.29	1.96 ± 0.32**	1.53 ± 0.19	1.35 ± 0.11**#**	1.57 ± 0.38	1.86 ± 0.26
LPC(18:2/0:0)	1.00 ± 0.35	2.11 ± 0.41**	1.56 ± 0.22	1.23 ± 0.19**##**	1.56 ± 0.43	1.97 ± 0.37
LPC(18:0/0:0)	1.00 ± 0.37	3.59 ± 1.37*	3.24 ± 0.94	2.55 ± 1.09	2.01 ± 1.03	5.02 ± 0.97
PE(18:3/22:4)	1.00 ± 0.30	2.40 ± 0.44**	1.27 ± 0.08	1.34 ± 0.31	1.22 ± 0.29**#**	1.23 ± 0.42**#**
PE(24:1/24:1)	1.00 ± 0.32	1.84 ± 0.32*	0.80 ± 0.07	0.86 ± 1.13	0.53 ± 0.08**##**	0.60 ± 0.12**##**
CE(24:1)	1.00 ± 0.42	2.11 ± 0.25*	1.11 ± 0.11**#**	1.43 ± 0.14	0.98 ± 0.57**##**	1.02 ± 0.11**##**

The average peak area of potential biomarkers in the control group was set as 1. C, control group; M, model group; Y, benzbromarone group; CL, low dosage group; CM, medium dosage group; CH, high dosage group; ANOVA and nonparametric test were used for statistical analysis, **, *p <* 0.01 vs. control group. *, *p <* 0.05 vs. control group; ##, *p < 0*.01 vs. model group; #, *p < 0.*05 vs. model group

### Metabolic pathways related to potential biomarker

**Fig. 7:**
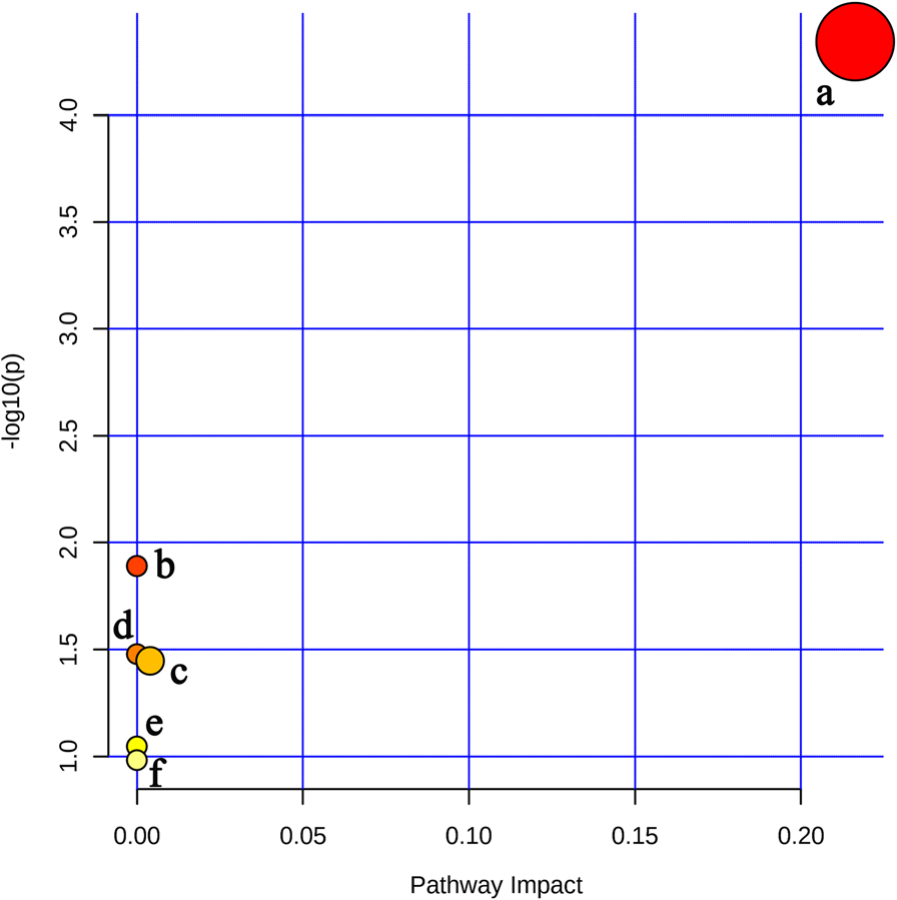
six pathway related to changed biomarkers. **a**: Glycerophospholipid metabolism, **b**: Linoleic acid metabolism, **c**: Glycosylphosphatidylinositol (GPI)-anchor biosynthesis, **d**: alpha-Linolenic acid metabolism, **e**: Arachidonic acid metabolism, f: Steroid biosynthesis

**Fig. 8:**
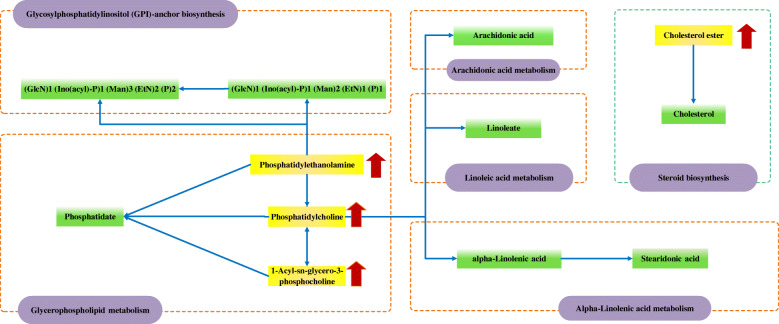
KEGG global metabolic network related to changed biomarkers. The purple textboxes represented the pathways, the yellow and green textboxes represented the significant and no detection metabolites. The arrows in red represented the up regulated metabolites. The arrows in blue represented direct or indirect connections between two metabolites

The 13 potential biomarkers were found to be primarily involved in 6 disturbed metabolic pathways. Based on the impact value greater than 0.1 and *p* value less than 0.05, glycerophospholipid metabolism was considered as the most relevant pathways in potassium oxonate-induced HUA (Fig. 7) and a global metabolic network was mapped (Fig. 8).

### Plantaginis Semen downregulated the mRNA expression of PI3k, Akt, URAT1 in HUA rats

**Fig. 9:**
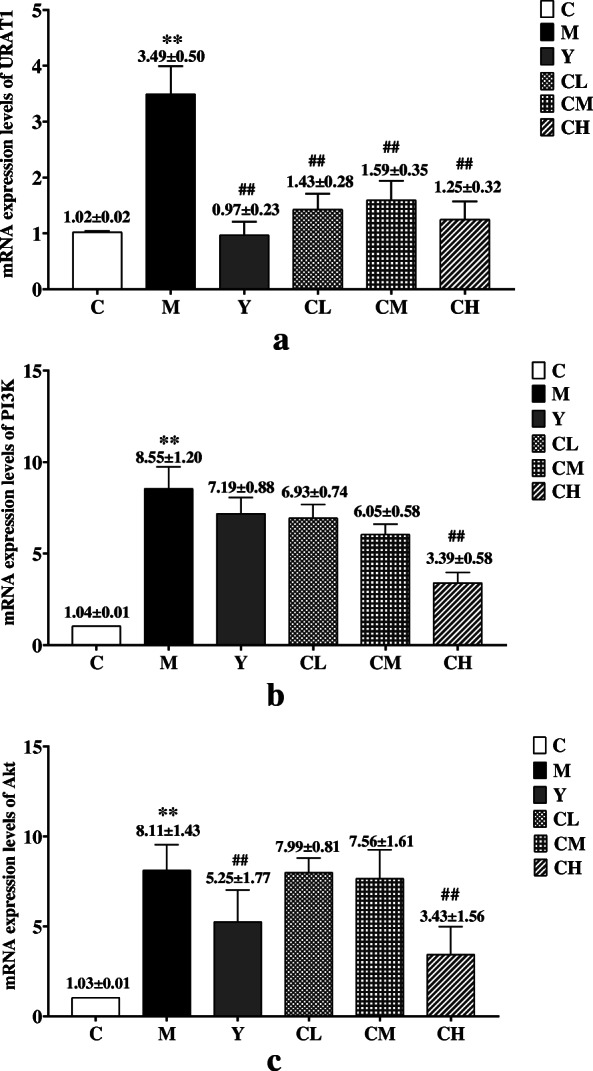
Effects of Plantaginis semen on PI3k/Akt and UTAR1 mRNA expression. **a** mRNA expression levels of URAT1, **b** mRNA expression levels of PI3k, **c** mRNA expression levels of Akt. C, control group; M, model group; Y, benzbromarone group; CL, low dosage group; CM, medium dosage group; CH, high dosage group; values are given as the mean ± SD(n = 7), ANOVA, Tukey post hoc and nonparametric test were used for statistical analysis, **, *P < 0.01* vs. control group. *, P < 0.05 vs. *control group; ##, P < 0.01* vs. *model group; #, P < 0.05* vs. *model group*

The mRNA expressions of PI3K, Akt and URAT1 in rat renal were shown in Fig. 9. The contents of URAT1 and PI3K/Akt were significantly (*P* < 0.01) increased in M group compared with those in C group. After 28 days administration, the Y group and three Plantaginis Semen groups all showed significantly (P < 0.01) down-regulation in the expressions of URAT1 mRNA, and the ability of inhibiting URATI mRNA expression among different Plantaginis Semen groups had no significantly differences (*p*>0.05, supplementary material, Table 5.4). In addition, only CH group could significantly (*P* < 0.01) decrease the mRNA expressions of PI3K and Akt in HUA rat, low and medium doses of Plantaginis Semen had no obvious inhibitory effect on the mRNA expression of PI3k and Akt, and there were no significant differences between the two groups (*p*>0.05; supplementary material, Table 6.3 and 7.4). The experimental results showed that the high dose Plantaginis Semen could inhibt the mRNA expressions of URAT1 and PI3K/Akt pathway.

## Discussion

Serum biochemical indicators were used to confirm the effects of Plantaginis Semen on the metabolism of lipid and anti-HUA. The serum UA levels of rats were significantly increased after potassium oxonate administration for 4 weeks. While increasing the serum UA of the rats, the levels of serum TNF-α, Cr and TG also increased. Compared with M group, different dose of Plantaginis Semen treatment could achieve varying degrees of down-regulation in the levers of serum UA in HUA rats and also reduced their serum Cr, TG and TNF-α concentration HUA is characterized by high serum UA level, urate crystals deposit in the blood vessel walls and kidneys that causes chronic inflammatory damage, releasing large amounts of inflammatory factors, such as TNF-α, IL-6 [40]. Plantaginis Semen polysaccharides ameliorated renal damage and decreased the inflammatory response in gouty nephropathy rats through the down-regulation of the protein expression levels of NLRP3, ASC and caspase-1 and inhibit the release of downstream inflammatory factors [41]. HUA is closely related to hyperlipidemia (HPA) and lipid metabolic disorder [42, 43], Lan JP et al. found that Plantaginis Semen had anti-obesity effects and could decrease serum TG and effectively improve lipid metabolism in high-fat diet-induced obese mice [44]. The results of serum biochemistry analysis in this study also suggested the elevated serum UA levels may contribute to the development and progression of chronic kidney disease, inflammatory and disordered lipid metabolism, Plantaginis Semen can alleviate the pathological state and exhibits hypouricemic, nephroprotective, anti-inflammation and regulation of lipid disorders effects.

UPLC-Q/TOF-MS-based Lipidomics was performed to discover distinct lipid metabolites and metabolic pathways related to HUA. 1 CE,2 TGs, 4 PCs, 4 LPCs, 2PEs were identified as biomarkers and glycerophospholipid metabolism pathway was mostly affected.

The results of lipidomics showed that the CE (24:1) content in the serum of HUA rats was significantly increased. The liver contains the most abundant cholesteryl ester hydrolase (CEH), so it is the main organ of cholesterol ester metabolism [45]. The abnormal improvement of serum CE (24:1) levels in HUA rats suggests that cholesterol ester metabolism in the liver may be affected under high serum UA environment. Under physiological conditions UA presents antioxidant properties, and HUA has been linked to oxidative stress, chronic low-grade inflammation, and insulin resistance, basic signs of non-alcoholic fatty liver disease (NAFLD) [20, 46]. HUA combined with NAFLD is the result of HUA progression, up-regulated phosphatidic acid and CE (18:0) and down-regulated inosine in the serum have been identified as the potential biomarkers for the progression from HUA to HUA + NAFLD [47]. In this study, we have also observed an upward tendency of CE (24:1). CE is involved in Liver X receptor/retinoic X receptor (LXR/RXR) activation, which plays an important role in keeping the cholesterol balance outside and inside the cell [48]. Given the results of lipidomics, liver may be preferentially targeted in HUA besides kidney and elevated serum UA levels may impair liver function and possibly act through LXR/RXR to influence cholesterol metabolism. In addition, high level of UA causes inflammation in the body, inflammatory stress exacerbates hepatic cholesterol accumulation via disrupting cellular cholesterol export [49], which further indicated that elevated UA level would lead to liver function damage and affected lipid metabolism. The level of CE (24:1) in Plantaginis Semen groups decreased, which domonstrated that Plantaginis Semen can protect liver and improve cholesterol metabolism. Genipidic acid contained in Plantaginis semen is the main component to exert these pharmacological action [50, 51].

Lipid metabolism disorder is closely related to HUA. HUA patients with HPA are typical in the clinical setting [52]. An investigation on the efficacy of Plantaginis Semen based on UPLC-QTOF-MS metabolomics approach in HPA mice showed Plantaginis Semen can improve blood lipids. In their study, serum levels of TG in HPA mice significantly increased, TG (16:1/16:0/o-18:0) was considered to be a potential biomarker and its content in the serum can be reduced by Plantaginis Semen [11]. Similarly, both An Peng et al. and Renhao Chen et al. found the level of TG increased in HUA model and glycerol tributanoate and Sn-glycerol-3P could be recognized as biomarker of HUA [53, 54]. In this study, M group had higher serum TG (18:2/18:1/18:2) and TG (20:4/14:0/18:3) levels than C group (M vs. C, *p* < 0.01), the treatment with Plantaginis Semen (at medium/high dose) effectively reduced the serum TG (18:2/18:1/18:2) levels of the HUA rats (CM/CH vs. M, *p* < 0.05). The results demonstrated that potassium oxonate disturbed lipid metabolism while Plantaginis Semen could accelerate the process of fat decomposition to alleviate lipid metabolism. Lipid-lowering therapy may provide a supplementary role to slow the development of HUA.

PC, LPC and PE were mainly involved in glycerophospholipids metabolism and their levels were obviously increased in HUA rats. LPCs are biosynthesized from PCs through glycerophospholipid metabolism, this process is catalyzed by cytoplasmic phospholipase A2 (PLA2) and can be activated by UA [55]. UA can activate the phospholipid-remodeling enzymes LPCAT3 in vivo and in vitro, and LPCAT3 possesses primary LPC acyltransferase activity and catalyzes the production of PCs [56]. The enhancement of PLA2 and LPCAT3 activity results in accelerated glycerophospholipid metabolism, so the levels of PC and LPC were both up-regulated in HUA rats. In our previous studies, PEs are key biomarkers of potassium oxonate induced HUA rats [57]. Interestingly, we also found that PE(18:3/22:4) and PE(24:1/24:1) are the key biomarkers in the treatment of HUA with Plantaginis Semen this time. In another study, the metabolism of phospholipids is seriously disturbed in the HUA mice and hydrolysis of glycerophospholipids are restrained, causing the up-regulation in the concentration of glycerophospholipids like PCs and PEs and the down-regulation in lysophospholipids and free fatty acid [58]. The results of the two studies are not completely consistent, the effect of HUA on PE metabolism needs to be further studied.

The results of lipidomics indicated that lipid metabolism disorder occurred in HUA rats, whereas, the changes were reversed under the Plantaginis Semen treatment. From a holistic perspective, variations in the metabolite profiles of different groups showed that Plantaginis Semen could enhance the metabolism of endogenous substances in HUA rats, which may be the potential mechanism of Plantaginis Semen in the treatment of HUA.

URAT1 is a key target for the treatment of HUA, and the metabolism of UA in kidney is completed by glomerular filtration, proximal tubule absorption and secretion, and mainly depends on the uric acid transporter to achieve [15]. The activation of PI3K/Akt pathway releases TNF-α, which plays an important role in inflammation caused by HUA [59]. Different doses of Plantaginis Semen all significantly inhibited the mRNA expression of URAT1 in the renal tissue of HUA rats and high dose of Plantaginis Semen possesses inhibitory activity of PI3K/Akt pathway. The results of PCR indicated that Plantaginis Semen may achieve the effect of treating HUA by regulating the expression level of URAT1, promoting uric acid excretion and reducing body inflammation. However, the mRNA level is not necessarily correlate with protein expression and the effects of Plantaginis Semen on URAT1 and PI3K/Akt pathway still needs to be further verified.

Although the toxicity of Plantaginis Semen has not been reported yet, Plantaginis Semen may have potential side effects due to its no obvious dose-dependent trend in this study. This section will be conducted in our future research. In summary, Plantaginis Semen has significant effects in reducing uric acid, protecting the kidneys and regulating lipid metabolism, our study exhibits its applicability and superiority in the treatment of HUA.

## Conclusions

Plantaginis Semen had significant anti-HUA, anti-inflammatory and renal protection effects and could attenuate potassium oxonate-induced HUA through regulation of lipid metabolism disorder. In addition, Plantaginis Semen could play anti-HUA effect through URAT1 and PI3K/Akt pathway, but the mechanism needs to be further studied.

## Supplementary Information

**Additional file 1.**

## Data Availability

The datasets used and/or analysed during the current study are available from the corresponding author on reasonable request.

## Abbreviations

ANOVA: One-way analysis of variance
CE: Cholesterol ester
CEH: Cholesteryl ester hydrolase
Cr: Creatinine
HUA: Hyperuricemia
HPA: Hyperlipidemia
PLA2: Cytoplasmic phospholipase A2
LPC: Hemolytic phosphatidylcholine
NAFLD: Non-alcoholic fatty liver disease
OPLS-DA: Orthogonal partial least squares discriminant analysis
Ox-LDL: Oxidized low-density lipoprotein
PCA: Principal component analysis
PC: Phosphatidylcholine
PE: Phosphatidylethanolamine
PI3K/Akt: Phosphatidylinositol 3-kinase/ protein kinases B
RT: Retention time
RT-qPCR: Quantitative real-time polymerase chain reaction
SPF: Specific pathogen free
SD: Sprague-Dawley
TC: Total cholesterol
TG: Triacylglycerol
TNF-α: Tumor necrosis factor-α
UA: Uric acid
UPLC−Q-TOF/MS: Ultra performance liquid chromatography quadrupole time of flight mass spectrometry
URAT1: urate anion transporter 1
VIP: Variable importance in the projection
XOD: Xanthine oxidase
