# Supplementary material for: Lipidomics study of the therapeutic mechanism of Plantaginis Semen in potassium oxonate-induced hyperuricemia rat
Source: BMC Complement Med Ther. 2021 Jun 25;21:175. doi: 10.1186/s12906-021-03350-x (PMC8235650; doi:10.1186/s12906-021-03350-x)
Supplement: Supplementary file 1 — Additional file 1. [file 12906_2021_3350_MOESM1_ESM.docx]

Table1.1 Results of normality test of serum UA level in each group

|  | Group | Shapiro-Wilk test | | | Result |
| --- | --- | --- | --- | --- | --- |
|  |  | Statistic | df | p-value |  |
| serum UA | C | 0.964 | 7 | 0.849 | normal distribution |
|  | M | 0.922 | 7 | 0.487 | normal distribution |
|  | Y | 0.932 | 7 | 0.564 | normal distribution |
|  | CL | 0.917 | 7 | 0.447 | normal distribution |
|  | CM | 0.895 | 7 | 0.304 | normal distribution |
|  | CH | 0.976 | 7 | 0.940 | normal distribution |

Table1.2 Results of homogeneity of variance test of serum UA level in each group

|  | | Levene statistic | df_1_ | df_2_ | p-value | Result |
| --- | --- | --- | --- | --- | --- | --- |
| serum UA | Based on the average | 1.346 | 5 | 36 | 0.267 | Samples have equal variance |

Table1.3 Results of ANOVA of serum UA level in each group

|  | Sum of squares | df | The mean square | F | p-value | Result |
| --- | --- | --- | --- | --- | --- | --- |
| between group variable | 27049.846 | 5 | 5409.969 | 38.074 | 0.000 | The mean values of each group are not all equal |
| interclass variance | 5115.236 | 36 | 142.090 |  |  |  |
| sum | 32165.082 | 41 |  |  |  |  |

Table1.4 Results of Tukey test of serum UA level in each group

| (I) Group | (J) Group | The mean difference  (I-J) | Standard error | p-value | 95% Confidence intervals | |
| --- | --- | --- | --- | --- | --- | --- |
|  |  |  |  |  | lower limit | upper limit |
| C | M | -82.87000^*^ | 6.37159 | 0.000 | -102.0394 | -63.7006 |
|  | Y | -41.90714^*^ | 6.37159 | 0.000 | -61.0765 | -22.7378 |
|  | CL | -53.66429^*^ | 6.37159 | 0.000 | -72.8337 | -34.4949 |
|  | CM | -64.06857^*^ | 6.37159 | 0.000 | -83.2380 | -44.8992 |
|  | CH | -43.82857^*^ | 6.37159 | 0.000 | -62.9980 | -24.6592 |
| M | C | 82.87000^*^ | 6.37159 | 0.000 | 63.7006 | 102.0394 |
|  | Y | 40.96286^*^ | 6.37159 | 0.000 | 21.7935 | 60.1322 |
|  | CL | 29.20571^*^ | 6.37159 | 0.001 | 10.0363 | 48.3751 |
|  | CM | 18.80143 | 6.37159 | 0.057 | -0.3680 | 37.9708 |
|  | CH | 39.04143^*^ | 6.37159 | 0.000 | 19.8720 | 58.2108 |
| Y | C | 41.90714^*^ | 6.37159 | 0.000 | 22.7378 | 61.0765 |
|  | M | -40.96286^*^ | 6.37159 | 0.000 | -60.1322 | -21.7935 |
|  | CL | -11.75714 | 6.37159 | 0.451 | -30.9265 | 7.4122 |
|  | CM | -22.16143^*^ | 6.37159 | 0.016 | -41.3308 | -2.9920 |
|  | CH | -1.92143 | 6.37159 | 1.000 | -21.0908 | 17.2480 |
| CL | C | 53.66429^*^ | 6.37159 | 0.000 | 34.4949 | 72.8337 |
|  | M | -29.20571^*^ | 6.37159 | 0.001 | -48.3751 | -10.0363 |
|  | Y | 11.75714 | 6.37159 | 0.451 | -7.4122 | 30.9265 |
|  | CM | -10.40429 | 6.37159 | 0.583 | -29.5737 | 8.7651 |
|  | CH | 9.83571 | 6.37159 | 0.639 | -9.3337 | 29.0051 |
| CM | C | 64.06857^*^ | 6.37159 | 0.000 | 44.8992 | 83.2380 |
|  | M | -18.80143 | 6.37159 | 0.057 | -37.9708 | 0.3680 |
|  | Y | 22.16143^*^ | 6.37159 | 0.016 | 2.9920 | 41.3308 |
|  | CL | 10.40429 | 6.37159 | 0.583 | -8.7651 | 29.5737 |
|  | CH | 20.24000^*^ | 6.37159 | 0.033 | 1.0706 | 39.4094 |
| CH | C | 43.82857^*^ | 6.37159 | 0.000 | 24.6592 | 62.9980 |
|  | M | -39.04143^*^ | 6.37159 | 0.000 | -58.2108 | -19.8720 |
|  | Y | 1.92143 | 6.37159 | 1.000 | -17.2480 | 21.0908 |
|  | CL | -9.83571 | 6.37159 | 0.639 | -29.0051 | 9.3337 |
|  | CM | -20.24000^*^ | 6.37159 | 0.033 | -39.4094 | -1.0706 |
| *: The significance level of mean difference was 0.05 | | | | | | |

Table2.1 Results of normality test of serum Cr level in each group

|  | Group | Shapiro-Wilk test | | | Result |
| --- | --- | --- | --- | --- | --- |
|  |  | Statistic | df | p-value |  |
| serum Cr | C | 0.918 | 7 | 0.458 | normal distribution |
|  | M | 0.849 | 7 | 0.120 | normal distribution |
|  | Y | 0.928 | 7 | 0.530 | normal distribution |
|  | CL | 0.910 | 7 | 0.396 | normal distribution |
|  | CM | 0.960 | 7 | 0.822 | normal distribution |
|  | CH | 0.973 | 7 | 0.921 | normal distribution |

Table2.2 Results of homogeneity of variance test of serum Cr level in each group

|  | | Levene statistic | df_1_ | df_2_ | p-value | Result |
| --- | --- | --- | --- | --- | --- | --- |
| serum Cr | Based on the average | 0.358 | 5 | 36 | 0.874 | Samples have equal variance |

Table2.3 Results of ANOVA of serum Cr level in each group

|  | Sum of squares | df | The mean square | F | p-value | Result |
| --- | --- | --- | --- | --- | --- | --- |
| between group variable | 683.668 | 5 | 136.734 | 7.748 | 0.000 | The mean values of each group are not all equal |
| interclass variance | 635.287 | 36 | 17.647 |  |  |  |
| sum | 1318.955 | 41 |  |  |  |  |

Table2.4 Results of Tukey test of serum Cr level in each group

| (I) Group | (J) Group | The mean difference  (I-J) | Standard error | p-value | 95% Confidence intervals | |
| --- | --- | --- | --- | --- | --- | --- |
|  |  |  |  |  | lower limit | upper limit |
| C | M | -11.42714^*^ | 2.24543 | 0.000 | -18.1827 | -4.6716 |
|  | Y | -9.11571^*^ | 2.24543 | 0.003 | -15.8713 | -2.3602 |
|  | CL | -5.05714 | 2.24543 | 0.240 | -11.8127 | 1.6984 |
|  | CM | -4.39714 | 2.24543 | 0.385 | -11.1527 | 2.3584 |
|  | CH | -1.21857 | 2.24543 | 0.994 | -7.9741 | 5.5370 |
| M | C | 11.42714^*^ | 2.24543 | 0.000 | 4.6716 | 18.1827 |
|  | Y | 2.31143 | 2.24543 | 0.905 | -4.4441 | 9.0670 |
|  | CL | 6.37000 | 2.24543 | 0.074 | -0.3855 | 13.1255 |
|  | CM | 7.03000^*^ | 2.24543 | 0.037 | 0.2745 | 13.7855 |
|  | CH | 10.20857^*^ | 2.24543 | 0.001 | 3.4530 | 16.9641 |
| Y | C | 9.11571^*^ | 2.24543 | 0.003 | 2.3602 | 15.8713 |
|  | M | -2.31143 | 2.24543 | 0.905 | -9.0670 | 4.4441 |
|  | CL | 4.05857 | 2.24543 | 0.474 | -2.6970 | 10.8141 |
|  | CM | 4.71857 | 2.24543 | 0.309 | -2.0370 | 11.4741 |
|  | CH | 7.89714^*^ | 2.24543 | 0.014 | 1.1416 | 14.6527 |
| CL | C | 5.05714 | 2.24543 | 0.240 | -1.6984 | 11.8127 |
|  | M | -6.37000 | 2.24543 | 0.074 | -13.1255 | 0.3855 |
|  | Y | -4.05857 | 2.24543 | 0.474 | -10.8141 | 2.6970 |
|  | CM | .66000 | 2.24543 | 1.000 | -6.0955 | 7.4155 |
|  | CH | 3.83857 | 2.24543 | 0.535 | -2.9170 | 10.5941 |
| CM | C | 4.39714 | 2.24543 | 0.385 | -2.3584 | 11.1527 |
|  | M | -7.03000^*^ | 2.24543 | 0.037 | -13.7855 | -0.2745 |
|  | Y | -4.71857 | 2.24543 | 0.309 | -11.4741 | 2.0370 |
|  | CL | -0.66000 | 2.24543 | 1.000 | -7.4155 | 6.0955 |
|  | CH | 3.17857 | 2.24543 | 0.718 | -3.5770 | 9.9341 |
| CH | C | 1.21857 | 2.24543 | 0.994 | -5.5370 | 7.9741 |
|  | M | -10.20857^*^ | 2.24543 | 0.001 | -16.9641 | -3.4530 |
|  | Y | -7.89714^*^ | 2.24543 | 0.014 | -14.6527 | -1.1416 |
|  | CL | -3.83857 | 2.24543 | 0.535 | -10.5941 | 2.9170 |
|  | CM | -3.17857 | 2.24543 | 0.718 | -9.9341 | 3.5770 |
| *: The significance level of mean difference was 0.05 | | | | | | |

Table 3.1 Results of normality test of serum TG level in each group

|  | Group | Shapiro-Wilk test | | | Result |
| --- | --- | --- | --- | --- | --- |
|  |  | Statistic | df | p-value |  |
| serum TG | C | 0.967 | 7 | 0.875 | normal distribution |
|  | M | 0.878 | 7 | 0.217 | normal distribution |
|  | Y | 0.953 | 7 | 0.759 | normal distribution |
|  | CL | 0.857 | 7 | 0.141 | normal distribution |
|  | CM | 0.981 | 7 | 0.964 | normal distribution |
|  | CH | 0.891 | 7 | 0.281 | normal distribution |

Table3.2 Results of homogeneity of variance test of serum TG level in each group

|  | | Levene statistic | df_1_ | df_2_ | p-value | Result |
| --- | --- | --- | --- | --- | --- | --- |
| serum TG | Based on the average | 2.187 | 5 | 36 | 0.077 | Samples have equal variance |

Table3.3 Results of ANOVA of serum TG level in each group

|  | Sum of squares | df | The mean square | F | p-value | Result |
| --- | --- | --- | --- | --- | --- | --- |
| between group variable | 0.615 | 5 | 0.123 | 14.242 | 0.000 | The mean values of each group are not all equal |
| interclass variance | 0.311 | 36 | 0.009 |  |  |  |
| sum | 0.927 | 41 |  |  |  |  |

Table3.4 Results of Tukey test of serum TG level in each group

| (I) Group | (J) Group | The mean difference  (I-J) | Standard error | p-value | 95% Confidence intervals | |
| --- | --- | --- | --- | --- | --- | --- |
|  |  |  |  |  | lower limit | upper limit |
| C | M | -0.36429^*^ | 0.04969 | 0.000 | -0.5138 | -0.2148 |
|  | Y | -0.10571 | 0.04969 | 0.297 | -0.2552 | 0.0438 |
|  | CL | -0.09857 | 0.04969 | 0.371 | -0.2481 | 0.0509 |
|  | CM | -0.03143 | 0.04969 | 0.988 | -0.1809 | 0.1181 |
|  | CH | -0.04000 | 0.04969 | 0.965 | -0.1895 | 0.1095 |
| M | C | 0.36429^*^ | 0.04969 | 0.000 | 0.2148 | 0.5138 |
|  | Y | 0.25857^*^ | 0.04969 | 0.000 | 0.1091 | 0.4081 |
|  | CL | 0.26571^*^ | 0.04969 | 0.000 | 0.1162 | 0.4152 |
|  | CM | 0.33286^*^ | 0.04969 | 0.000 | 0.1834 | 0.4824 |
|  | CH | 0.32429^*^ | 0.04969 | 0.000 | 0.1748 | 0.4738 |
| Y | C | 0.10571 | 0.04969 | 0.297 | -0.0438 | 0.2552 |
|  | M | -0.25857^*^ | 0.04969 | 0.000 | -0.4081 | -0.1091 |
|  | CL | 0.00714 | 0.04969 | 1.000 | -0.1424 | 0.1566 |
|  | CM | 0.07429 | 0.04969 | 0.670 | -0.0752 | 0.2238 |
|  | CH | 0.06571 | 0.04969 | 0.771 | -0.0838 | 0.2152 |
| CL | C | 0.09857 | 0.04969 | 0.371 | -0.0509 | 0.2481 |
|  | M | -0.26571^*^ | 0.04969 | 0.000 | -0.4152 | -0.1162 |
|  | Y | -0.00714 | 0.04969 | 1.000 | -0.1566 | 0.1424 |
|  | CM | 0.06714 | 0.04969 | 0.755 | -0.0824 | 0.2166 |
|  | CH | 0.05857 | 0.04969 | 0.844 | -0.0909 | 0.2081 |
| CM | C | 0.03143 | 0.04969 | 0.988 | -0.1181 | 0.1809 |
|  | M | -0.33286^*^ | 0.04969 | 0.000 | -0.4824 | -0.1834 |
|  | Y | -0.07429 | 0.04969 | 0.670 | -0.2238 | 0.0752 |
|  | CL | -0.06714 | 0.04969 | 0.755 | -0.2166 | 0.0824 |
|  | CH | -0.00857 | 0.04969 | 1.000 | -0.1581 | 0.1409 |
| CH | C | 0.04000 | 0.04969 | 0.965 | -0.1095 | 0.1895 |
|  | M | -0.32429^*^ | 0.04969 | 0.000 | -0.4738 | -0.1748 |
|  | Y | -0.06571 | 0.04969 | 0.771 | -0.2152 | 0.0838 |
|  | CL | -0.05857 | 0.04969 | 0.844 | -0.2081 | 0.0909 |
|  | CM | 0.00857 | 0.04969 | 1.000 | -0.1409 | 0.1581 |
| *: The significance level of mean difference was 0.05 | | | | | | |

Table 4.1 Results of normality test of TNF-α level in each group

|  | Group | Shapiro-Wilk test | | | Result |
| --- | --- | --- | --- | --- | --- |
|  |  | Statistic | df | p-value |  |
| TNF-α | C | 0.897 | 7 | 0.316 | normal distribution |
|  | M | 0.872 | 7 | 0.193 | normal distribution |
|  | Y | 0.976 | 7 | 0.936 | normal distribution |
|  | CL | 0.830 | 7 | 0.079 | normal distribution |
|  | CM | 0.988 | 7 | 0.990 | normal distribution |
|  | CH | 0.897 | 7 | 0.316 | normal distribution |

Table4.2 Results of homogeneity of variance test of TNF-α level in each group

|  | | Levene statistic | df_1_ | df_2_ | p-value | Result |
| --- | --- | --- | --- | --- | --- | --- |
| TNF-α | Based on the average | 2.087 | 5 | 36 | 0.090 | Samples have equal variance |

Table4.3 Results of ANOVA of TNF-α level in each group

|  | Sum of squares | df | The mean square | F | p-value | Result |
| --- | --- | --- | --- | --- | --- | --- |
| between group variable | 12021.151 | 5 | 2404.230 | 9.483 | .000 | The mean values of each group are not all equal |
| interclass variance | 9126.883 | 36 | 253.525 |  |  |  |
| sum | 21148.034 | 41 |  |  |  |  |

Table4.4 Results of Tukey test of TNF-α level in each group

| (I) Group | (J) Group | The mean difference  (I-J) | Standard error | p-value | 95% Confidence intervals | |
| --- | --- | --- | --- | --- | --- | --- |
|  |  |  |  |  | lower limit | upper limit |
| C | M | -41.40286^*^ | 8.51091 | 0.000 | -67.0086 | -15.7972 |
|  | Y | 12.42714 | 8.51091 | 0.691 | -13.1786 | 38.0328 |
|  | CL | 0.47286 | 8.51091 | 1.000 | -25.1328 | 26.0786 |
|  | CM | -9.13429 | 8.51091 | 0.889 | -34.7400 | 16.4714 |
|  | CH | -15.14286 | 8.51091 | 0.491 | -40.7486 | 10.4628 |
| M | C | 41.40286^*^ | 8.51091 | 0.000 | 15.7972 | 67.0086 |
|  | Y | 53.83000^*^ | 8.51091 | 0.000 | 28.2243 | 79.4357 |
|  | CL | 41.87571^*^ | 8.51091 | 0.000 | 16.2700 | 67.4814 |
|  | CM | 32.26857^*^ | 8.51091 | 0.007 | 6.6629 | 57.8743 |
|  | CH | 26.26000^*^ | 8.51091 | 0.042 | 0.6543 | 51.8657 |
| Y | C | -12.42714 | 8.51091 | 0.691 | -38.0328 | 13.1786 |
|  | M | -53.83000^*^ | 8.51091 | 0.000 | -79.4357 | -28.2243 |
|  | CL | -11.95429 | 8.51091 | 0.724 | -37.5600 | 13.6514 |
|  | CM | -21.56143 | 8.51091 | 0.141 | -47.1671 | 4.0443 |
|  | CH | -27.57000^*^ | 8.51091 | 0.029 | -53.1757 | -1.9643 |
| CL | C | -0.47286 | 8.51091 | 1.000 | -26.0786 | 25.1328 |
|  | M | -41.87571^*^ | 8.51091 | 0.000 | -67.4814 | -16.2700 |
|  | Y | 11.95429 | 8.51091 | 0.724 | -13.6514 | 37.5600 |
|  | CM | -9.60714 | 8.51091 | 0.866 | -35.2128 | 15.9986 |
|  | CH | -15.61571 | 8.51091 | 0.457 | -41.2214 | 9.9900 |
| CM | C | 9.13429 | 8.51091 | .889 | -16.4714 | 34.7400 |
|  | M | -32.26857^*^ | 8.51091 | .007 | -57.8743 | -6.6629 |
|  | Y | 21.56143 | 8.51091 | .141 | -4.0443 | 47.1671 |
|  | CL | 9.60714 | 8.51091 | .866 | -15.9986 | 35.2128 |
|  | CH | -6.00857 | 8.51091 | .980 | -31.6143 | 19.5971 |
| CH | C | 15.14286 | 8.51091 | .491 | -10.4628 | 40.7486 |
|  | M | -26.26000^*^ | 8.51091 | .042 | -51.8657 | -.6543 |
|  | Y | 27.57000^*^ | 8.51091 | .029 | 1.9643 | 53.1757 |
|  | CL | 15.61571 | 8.51091 | .457 | -9.9900 | 41.2214 |
|  | CM | 6.00857 | 8.51091 | .980 | -19.5971 | 31.6143 |
| *: The significance level of mean difference was 0.05 | | | | | | |

Table5.1 Results of normality test of relative mRNA expression levels of URAT1 in each group

|  | Group | Shapiro-Wilk test | | | Result |
| --- | --- | --- | --- | --- | --- |
|  |  | Statistic | df | p-value |  |
| URAT1 mRNA | C | 0.840 | 7 | 0.100 | normal distribution |
|  | M | 0.863 | 7 | 0.161 | normal distribution |
|  | Y | 0.841 | 7 | 0.100 | normal distribution |
|  | CL | 0.987 | 7 | 0.987 | normal distribution |
|  | CM | 0.900 | 7 | 0.334 | normal distribution |
|  | CH | 0.925 | 7 | 0.510 | normal distribution |

Table5.2 Results of homogeneity of variance test of mRNA expression levels of URAT1 in each group

|  | | Levene statistic | df_1_ | df_2_ | p-value | Result |
| --- | --- | --- | --- | --- | --- | --- |
| URAT1 mRNA | Based on the average | 1.791 | 5 | 36 | 0.139 | Samples have equal variance |

Table5.3 Results of ANOVA of mRNA expression levels of URAT1 in each group

|  | Sum of squares | df | The mean square | F | p-value | Result |
| --- | --- | --- | --- | --- | --- | --- |
| between group variable | 31.338 | 5 | 6.268 | 61.192 | 0.000 | The mean values of each group are not all equal |
| interclass variance | 3.687 | 36 | 0.102 |  |  |  |
| sum | 35.025 | 41 |  |  |  |  |

Table5.4 Results of Tukey test of mRNA expression levels of URAT1 in each group

| (I) Group | (J) Group | The mean difference  (I-J) | Standard error | p-value | 95% Confidence intervals | |
| --- | --- | --- | --- | --- | --- | --- |
|  |  |  |  |  | lower limit | upper limit |
| C | M | -2.47286^*^ | 0.17107 | 0.000 | -2.9875 | -1.9582 |
|  | Y | 0.05571 | 0.17107 | 0.999 | -0.4590 | 0.5704 |
|  | CL | -0.40429 | 0.17107 | 0.196 | -0.9190 | 0.1104 |
|  | CM | -0.57000^*^ | 0.17107 | 0.023 | -1.0847 | -.0553 |
|  | CH | -0.22571 | 0.17107 | 0.772 | -0.7404 | 0.2890 |
| M | C | 2.47286^*^ | 0.17107 | 0.000 | 1.9582 | 2.9875 |
|  | Y | 2.52857^*^ | 0.17107 | 0.000 | 2.0139 | 3.0432 |
|  | CL | 2.06857^*^ | 0.17107 | 0.000 | 1.5539 | 2.5832 |
|  | CM | 1.90286^*^ | 0.17107 | 0.000 | 1.3882 | 2.4175 |
|  | CH | 2.24714^*^ | 0.17107 | 0.000 | 1.7325 | 2.7618 |
| Y | C | -0.05571 | 0.17107 | 0.999 | -.5704 | 0.4590 |
|  | M | -2.52857^*^ | 0.17107 | 0.000 | -3.0432 | -2.0139 |
|  | CL | -0.46000 | 0.17107 | 0.102 | -.9747 | 0.0547 |
|  | CM | -0.62571^*^ | 0.17107 | 0.010 | -1.1404 | -.01110 |
|  | CH | -0.28143 | 0.17107 | 0.575 | -.7961 | 0.2332 |
| CL | C | 0.40429 | 0.17107 | 0.196 | -.1104 | 0.9190 |
|  | M | -2.06857^*^ | 0.17107 | 0.000 | -2.5832 | -1.5539 |
|  | Y | 0.46000 | 0.17107 | 0.102 | -.0547 | 0.9747 |
|  | CM | -0.16571 | 0.17107 | 0.925 | -.6804 | 0.3490 |
|  | CH | 0.17857 | 0.17107 | 0.900 | -.3361 | 0.6932 |
| CM | C | 0.57000^*^ | 0.17107 | 0.023 | 0.0553 | 1.0847 |
|  | M | -1.90286^*^ | 0.17107 | 0.000 | -2.4175 | -1.3882 |
|  | Y | 0.62571^*^ | 0.17107 | 0.010 | 0.1110 | 1.1404 |
|  | CL | 0.16571 | 0.17107 | 0.925 | -.3490 | 0.6804 |
|  | CH | 0.34429 | 0.17107 | 0.355 | -.1704 | 0.8590 |
| CH | C | 0.22571 | 0.17107 | 0.772 | -.2890 | 0.7404 |
|  | M | -2.24714^*^ | 0.17107 | 0.000 | -2.7618 | -1.7325 |
|  | Y | 0.28143 | 0.17107 | 0.575 | -.2332 | 0.7961 |
|  | CL | -.017857 | 0.17107 | 0.900 | -.6932 | 0.3361 |
|  | CM | -0.34429 | 0.17107 | 0.355 | -.8590 | 0.1704 |
| *: The significance level of mean difference was 0.05 | | | | | | |

Table6.1 Results of normality test of relative mRNA expression levels of PI3k in each group

|  | Group | Shapiro-Wilk test | | | Result |
| --- | --- | --- | --- | --- | --- |
|  |  | Statistic | df | p-value |  |
| PI3k mRNA | C | 0.937 | 7 | 0.609 | normal distribution |
|  | M | 0.841 | 7 | 0.100 | normal distribution |
|  | Y | 0.926 | 7 | 0.520 | normal distribution |
|  | CL | 0.826 | 7 | 0.073 | normal distribution |
|  | CM | 0.859 | 7 | 0.149 | normal distribution |
|  | CH | 0.931 | 7 | 0.560 | normal distribution |

Table6.2 Results of homogeneity of variance test of mRNA expression levels of PI3k in each group

|  | | Levene statistic | df_1_ | df_2_ | p-value | Result |
| --- | --- | --- | --- | --- | --- | --- |
| PI3k mRNA | Based on the average | 5.012 | 5 | 36 | 0.001 | The variance of samples is not equal |

Table6.3 The nonparametric tests results of mRNA expression levels of PI3k

| (I) Group | (J) Group | p-value |
| --- | --- | --- |
| C | M | 0.000 |
|  | Y | 0.001 |
|  | CL | 0.005 |
|  | CM | 0.220 |
|  | CH | 1.000 |
| M | C | 0.000 |
|  | Y | 1.000 |
|  | CL | 1.000 |
|  | CM | 0.152 |
|  | CH | 0.001 |
| Y | C | 0.001 |
|  | M | 1.000 |
|  | CL | 1.000 |
|  | CM | 1.000 |
|  | CH | 0.065 |
| CL | C | 0.005 |
|  | M | 1.000 |
|  | Y | 1.000 |
|  | CM | 1.000 |
|  | CH | 0.183 |
| CM | C | 0.220 |
|  | M | 0.152 |
|  | Y | 1.000 |
|  | CL | 1.000 |
|  | CH | 1.000 |
| CH | C | 1.000 |
|  | M | 0.001 |
|  | Y | 0.065 |
|  | CL | 0.183 |
|  | CM | 1.000 |

Table 7.1 Results of normality test of relative mRNA expression levels of Akt in each group

|  | Group | Shapiro-Wilk test | | | Result |
| --- | --- | --- | --- | --- | --- |
|  |  | Statistic | df | p-value |  |
| Akt  mRNA | C | .869 | 7 | .183 | normal distribution |
|  | M | .937 | 7 | .610 | normal distribution |
|  | Y | .964 | 7 | .850 | normal distribution |
|  | CL | .816 | 7 | .059 | normal distribution |
|  | CM | .907 | 7 | .377 | normal distribution |
|  | CH | .935 | 7 | .594 | normal distribution |

Table7.2 Results of homogeneity of variance test of mRNA expression levels of Akt in each group

|  | | Levene statistic | df_1_ | df_2_ | p-value | Result |
| --- | --- | --- | --- | --- | --- | --- |
| Akt  mRNA | Based on the average | 2.249 | 5 | 36 | 0.070 | Samples have equal variance |

Table7.3 Results of ANOVA of mRNA expression levels of Akt in each group

|  | Sum of squares | df | The mean square | F | p-value | Result |
| --- | --- | --- | --- | --- | --- | --- |
| between group variable | 293.446 | 5 | 58.693 | 32.527 | .000 | The mean values of each group are not all equal |
| interclass variance | 69.960 | 36 | 1.840 |  |  |  |
| sum | 358.426 | 41 |  |  |  |  |

Table7.4 Results of Tukey test of mRNA expression levels of Akt in each group

| (I) Group | (J) Group | The mean difference  (I-J) | Standard error | p-value | 95% Confidence intervals | |
| --- | --- | --- | --- | --- | --- | --- |
|  |  |  |  |  | lower limit | upper limit |
| C | M | -7.07882^*^ | .71802 | .000 | -9.2390 | -4.9186 |
|  | Y | -4.21424^*^ | .71802 | .000 | -6.3745 | -2.0540 |
|  | CL | -6.96165^*^ | .71802 | .000 | -9.1219 | -4.8014 |
|  | CM | -6.61708^*^ | .71802 | .000 | -8.7773 | -4.4569 |
|  | CH | -2.39946^*^ | .71802 | .022 | -4.5597 | -.2392 |
| M | C | 7.07882^*^ | .71802 | .000 | 4.9186 | 9.2390 |
|  | Y | 2.86459^*^ | .71802 | .004 | .7044 | 5.0248 |
|  | CL | .11717 | .71802 | 1.000 | -2.0430 | 2.2774 |
|  | CM | .46174 | .71802 | .987 | -1.6985 | 2.6220 |
|  | CH | 4.67936^*^ | .71802 | .000 | 2.5191 | 6.8396 |
| Y | C | 4.21424^*^ | .71802 | .000 | 2.0540 | 6.3745 |
|  | M | -2.86459^*^ | .71802 | .004 | -5.0248 | -.7044 |
|  | CL | -2.74741^*^ | .71802 | .006 | -4.9076 | -.5872 |
|  | CM | -2.40285^*^ | .71802 | .022 | -4.5631 | -.2426 |
|  | CH | 1.81478 | .71802 | .143 | -.3454 | 3.9750 |
| CL | C | 6.96165^*^ | .71802 | .000 | 4.8014 | 9.1219 |
|  | M | -.11717 | .71802 | 1.000 | -2.2774 | 2.0430 |
|  | Y | 2.74741^*^ | .71802 | .006 | .5872 | 4.9076 |
|  | CM | .34457 | .71802 | .997 | -1.8156 | 2.5048 |
|  | CH | 4.56219^*^ | .71802 | .000 | 2.4020 | 6.7224 |
| CM | C | 6.61708^*^ | .71802 | .000 | 4.4569 | 8.7773 |
|  | M | -.46174 | .71802 | .987 | -2.6220 | 1.6985 |
|  | Y | 2.40285^*^ | .71802 | .022 | .2426 | 4.5631 |
|  | CL | -.34457 | .71802 | .997 | -2.5048 | 1.8156 |
|  | CH | 4.21762^*^ | .71802 | .000 | 2.0574 | 6.3778 |
| CH | C | 2.39946^*^ | .71802 | .022 | .2392 | 4.5597 |
|  | M | -4.67936^*^ | .71802 | .000 | -6.8396 | -2.5191 |
|  | Y | -1.81478 | .71802 | .143 | -3.9750 | .3454 |
|  | CL | -4.56219^*^ | .71802 | .000 | -6.7224 | -2.4020 |
|  | CM | -4.21762^*^ | .71802 | .000 | -6.3778 | -2.0574 |
| *: The significance level of mean difference was 0.05 | | | | | | |

Table 8.1 The statistical test results of TG(18:2/18:1/18:2)

|  | normality test | | homogeneity of variance test | |
| --- | --- | --- | --- | --- |
| TG(18:2/18:1/18:2) | p-value | Result | p-value | Result |
|  | P_CM_=0.04 | Non-normal distribution | 0.000 | The variance of samples is not equal |

Table 8.2 The nonparametric tests results of TG(18:2/18:1/18:2)

| (I) Group | (J) Group | p-value |
| --- | --- | --- |
| C | M | 0.004 |
|  | Y | 1.000 |
|  | CL | 0.006 |
|  | CM | 1.000 |
|  | CH | 1.000 |
| M | C | 0.004 |
|  | Y | 0.749 |
|  | CL | 1.000 |
|  | CM | 0.022 |
|  | CH | 0.000 |

Table 9.1 The statistical test results of TG(20:4/14:0/18:3)

|  | normality test | | homogeneity of variance test | |
| --- | --- | --- | --- | --- |
| TG(20:4/14:0/18:3) | p-value | Result | p-value | Result |
|  | P＞0.05 | normal distribution | 0.001 | The variance of samples is not equal |

Table 9.2 The nonparametric tests results of TG(20:4/14:0/18:3)

| (I) Group | (J) Group | p-value |
| --- | --- | --- |
| C | M | 0.001 |
|  | Y | 0.143 |
|  | CL | 0.006 |
|  | CM | 1.000 |
|  | CH | 0.001 |
| M | C | 0.001 |
|  | Y | 1.000 |
|  | CL | 1.000 |
|  | CM | 0.220 |
|  | CH | 1.000 |

Table 10.1 The statistical test results of PC(24:1/16:1)

|  | normality test | | homogeneity of variance test | |
| --- | --- | --- | --- | --- |
| PC(24:1/16:1) | p-value | Result | p-value | Result |
|  | P＞0.05 | normal distribution | 0.000 | The variance of samples is not equal |

Table 10.2 The nonparametric tests results of PC(24:1/16:1)

| (I) Group | (J) Group | p-value |
| --- | --- | --- |
| C | M | 0.000 |
|  | Y | 0.046 |
|  | CL | 0.000 |
|  | CM | 1.000 |
|  | CH | 0.264 |
| M | C | 0.000 |
|  | Y | 1.000 |
|  | CL | 1.000 |
|  | CM | 0.011 |
|  | CH | 0.234 |

Table 11.1 The statistical test results of PC(24:1/18:2)

|  | normality test | | homogeneity of variance test | |
| --- | --- | --- | --- | --- |
| PC(24:1/18:2) | p-value | Result | p-value | Result |
|  | P＞0.05 | normal distribution | 0.000 | The variance of samples is not equal |

Table 11.2 The nonparametric tests results of PC(24:1/18:2)

| (I) Group | (J) Group | p-value |
| --- | --- | --- |
| C | M | 0.001 |
|  | Y | 0.002 |
|  | CL | 0.749 |
|  | CM | 1.000 |
|  | CH | 1.000 |
| M | C | 0.001 |
|  | Y | 1.000 |
|  | CL | 0.711 |
|  | CM | 0.001 |
|  | CH | 0.019 |

Table 12.1 The statistical test results of PC(15:0/18:0)

|  | normality test | | homogeneity of variance test | |
| --- | --- | --- | --- | --- |
| PC(15:0/18:0) | p-value | Result | p-value | Result |
|  | P＞0.05 | normal distribution | 0.006 | The variance of samples is not equal |

Table 12.2 The nonparametric tests results of PC(15:0/18:0)

| (I) Group | (J) Group | p-value |
| --- | --- | --- |
| C | M | 0.003 |
|  | Y | 0.065 |
|  | CL | 0.173 |
|  | CM | 1.000 |
|  | CH | 0.440 |
| M | C | 0.003 |
|  | Y | 1.000 |
|  | CL | 1.000 |
|  | CM | 0.034 |
|  | CH | 1.000 |

Table 13.1 The statistical test results of PC(24:1/22:2)

|  | normality test | | homogeneity of variance test | |
| --- | --- | --- | --- | --- |
| PC(24:1/22:2) | p-value | Result | p-value | Result |
|  | P_Y_=0.039 | Non-normal distribution | 0.001 | The variance of samples is not equal |

Table 13.2 The nonparametric tests results of PC(24:1/22:2)

| (I) Group | (J) Group | p-value |
| --- | --- | --- |
| C | M | 0.003 |
|  | Y | 1.000 |
|  | CL | 1.000 |
|  | CM | 0.046 |
|  | CH | 0.248 |
| M | C | 0.003 |
|  | Y | 0.010 |
|  | CL | 0.641 |
|  | CM | 0.000 |
|  | CH | 0.001 |

Table 14.1 The statistical test results of PC(24:1/22:2)

|  | normality test | | homogeneity of variance test | |
| --- | --- | --- | --- | --- |
| PC(24:1/22:2) | p-value | Result | p-value | Result |
|  | P＞0.05 | normal distribution | 0.033 | The variance of samples is not equal |

Table 14.2 The nonparametric tests results of PC(24:1/22:2)

| (I) Group | (J) Group | p-value |
| --- | --- | --- |
| C | M | 0.001 |
|  | Y | 0.314 |
|  | CL | 0.314 |
|  | CM | 0.352 |
|  | CH | 0.002 |
| M | C | 0.001 |
|  | Y | 1.000 |
|  | CL | 1.000 |
|  | CM | 1.000 |
|  | CH | 1.000 |

Table 15.1 The statistical test results of LPC(22:4/0:0)

|  | normality test | | homogeneity of variance test | | ANOVA | |
| --- | --- | --- | --- | --- | --- | --- |
| LysoPC(22:4/0:0) | p-value | Result | p-value | Result | p-value | Result |
|  | P＞0.05 | Non-normal distribution | 0.547 | Samples have equal variance | 0.000 | The mean values of each group are not all equal |

Table15.2 Results of Tukey test of LPC(22:4/0:0) levels in each group

| (I) Group | (J) Group | The mean difference  (I-J) | Standard error | p-value | 95% Confidence intervals | |
| --- | --- | --- | --- | --- | --- | --- |
|  |  |  |  |  | lower limit | upper limit |
| C | M | -.964286^*^ | 0.146831 | .000 | -1.40604 | -.52253 |
|  | Y | -.529429^*^ | 0.146831 | .011 | -.97118 | -.08768 |
|  | CL | -.348571 | 0.146831 | .192 | -.79032 | .09318 |
|  | CM | -.567429^*^ | 0.146831 | .006 | -1.00918 | -.12568 |
|  | CH | -.862857^*^ | 0.146831 | .000 | -1.30461 | -.42110 |
| M | C | .964286^*^ | 0.146831 | .000 | .52253 | 1.40604 |
|  | Y | .434857 | 0.146831 | .056 | -.00690 | .87661 |
|  | CL | .615714^*^ | 0.146831 | .002 | .17396 | 1.05747 |
|  | CM | .396857 | 0.146831 | .099 | -.04490 | .83861 |
|  | CH | .101429 | 0.146831 | .982 | -.34032 | .54318 |
| Y | C | .529429^*^ | 0.146831 | .011 | .08768 | .97118 |
|  | M | -.434857 | 0.146831 | .056 | -.87661 | .00690 |
|  | CL | .180857 | 0.146831 | .818 | -.26090 | .62261 |
|  | CM | -.038000 | 0.146831 | 1.000 | -.47975 | .40375 |
|  | CH | -.333429 | 0.146831 | .232 | -.77518 | .10832 |
| CL | C | .348571 | 0.146831 | .192 | -.09318 | .79032 |
|  | M | -.615714^*^ | 0.146831 | .002 | -1.05747 | -.17396 |
|  | Y | -.180857 | 0.146831 | .818 | -.62261 | .26090 |
|  | CM | -.218857 | 0.146831 | .672 | -.66061 | .22290 |
|  | CH | -.514286^*^ | 0.146831 | .015 | -.95604 | -.07253 |
| CM | C | .567429^*^ | 0.146831 | .006 | .12568 | 1.00918 |
|  | M | -.396857 | 0.146831 | .099 | -.83861 | .04490 |
|  | Y | .038000 | 0.146831 | 1.000 | -.40375 | .47975 |
|  | CL | .218857 | 0.146831 | .672 | -.22290 | .66061 |
|  | CH | -.295429 | 0.146831 | .356 | -.73718 | .14632 |
| CH | C | .862857^*^ | 0.146831 | .000 | .42110 | 1.30461 |
|  | M | -.101429 | 0.146831 | .982 | -.54318 | .34032 |
|  | Y | .333429 | 0.146831 | .232 | -.10832 | .77518 |
|  | CL | .514286^*^ | 0.146831 | .015 | .07253 | .95604 |
|  | CM | .295429 | 0.146831 | .356 | -.14632 | .73718 |
| *: The significance level of mean difference was 0.05 | | | | | | |

Table 16.1 The statistical test results of LPC(18:2/0:0)

|  | normality test | | homogeneity of variance test | |
| --- | --- | --- | --- | --- |
| LysoPC(18:2/0:0) | p-value | Result | p-value | Result |
|  | P_C_=0.029 | Non-normal distribution | 0.596 | Samples have equal variance |

Table 16.2 The nonparametric tests results of PC(24:1/22:2)

| (I) Group | (J) Group | p-value |
| --- | --- | --- |
| C | M | 0.000 |
|  | Y | 0.234 |
|  | CL | 1.000 |
|  | CM | 0.417 |
|  | CH | 0.002 |
| M | C | 0.000 |
|  | Y | 0.871 |
|  | CL | 0.008 |
|  | CM | 0.519 |
|  | CH | 1.000 |

Table 17.1 The statistical test results of LPC(18:0/0:0)

|  | normality test | | homogeneity of variance test | |
| --- | --- | --- | --- | --- |
| LysoPC(18:0/0:0) | p-value | Result | p-value | Result |
|  | P＞0.05 | normal distribution | 0.029 | The variance of samples is not equal |

Table 17.2 The nonparametric tests results of LysoPC(18:0/0:0)

| (I) Group | (J) Group | p-value |
| --- | --- | --- |
| C | M | 0.019 |
|  | Y | 0.030 |
|  | CL | 0.440 |
|  | CM | 1.000 |
|  | CH | 0.000 |
| M | C | 0.019 |
|  | Y | 1.000 |
|  | CL | 1.000 |
|  | CM | 1.000 |
|  | CH | 1.000 |

Table 18.1 The statistical test results of PE(18:3/22:4)

|  | normality test | | homogeneity of variance test | |
| --- | --- | --- | --- | --- |
| PE(18:3/22:4) | p-value | Result | p-value | Result |
|  | P＞0.05 | normal distribution | 0.038 | The variance of samples is not equal |

Table 18.2 The nonparametric tests results of PE(18:3/22:4)

| (I) Group | (J) Group | p-value |
| --- | --- | --- |
| C | M | 0.000 |
|  | Y | 1.000 |
|  | CL | 1.000 |
|  | CM | 1.000 |
|  | CH | 1.000 |
| M | C | 0.000 |
|  | Y | 0.053 |
|  | CL | 0.134 |
|  | CM | 0.032 |
|  | CH | 0.024 |

Table 19.1 The statistical test results of PE(24:1/24:1)

|  | normality test | | homogeneity of variance test | |
| --- | --- | --- | --- | --- |
| (24:1/24:1) | p-value | Result | p-value | Result |
|  | P_C_=0.029 | Non-normal distribution | 0.596 | Samples have equal variance |

Table 19.2 The nonparametric tests results of PE(24:1/24:1)

| (I) Group | (J) Group | p-value |
| --- | --- | --- |
| C | M | 0.022 |
|  | Y | 1.000 |
|  | CL | 1.000 |
|  | CM | 0.038 |
|  | CH | 0.256 |
| M | C | 0.022 |
|  | Y | 0.263 |
|  | CL | 0.465 |
|  | CM | 0.000 |
|  | CH | 0.000 |

Table 20.1 The statistical test results of CE(24:1)

|  | normality test | | homogeneity of variance test | |
| --- | --- | --- | --- | --- |
| CE(24:1) | p-value | Result | p-value | Result |
|  | P＞0.05 | normal distribution | 0.000 | The variance of samples is not equal |

Table 20.2 The nonparametric tests results of CE(24:1)

| (I) Group | (J) Group | p-value |
| --- | --- | --- |
| C | M | 0.030 |
|  | Y | 1.000 |
|  | CL | 0.961 |
|  | CM | 1.000 |
|  | CH | 1.000 |
| M | C | 0.030 |
|  | Y | 0.026 |
|  | CL | 1.000 |
|  | CM | 0.000 |
|  | CH | 0.001 |
